# Chemical feedbacks during magma degassing control chlorine partitioning and metal extraction in volcanic arcs

**DOI:** 10.1038/s41467-021-21887-w

**Published:** 2021-03-19

**Authors:** B. Tattitch, C. Chelle-Michou, J. Blundy, R. R. Loucks

**Affiliations:** 1 School of Earth Sciences, University of Bristol, Bristol, UK; 2 Institute of Geochemistry and Petrology, Department of Earth Sciences, ETH Zürich, Zürich, Switzerland; 3 Department of Earth Sciences, University of Oxford, Oxford, UK; 4 Centre for Exploration Targeting, School of Earth and Environment, University of Western Australia, Crawley, WA Australia

**Keywords:** Geochemistry, Petrology, Volcanology

## Abstract

Hydrous fluids released from subducting oceanic lithosphere fuel arc magmatism and associated hydrothermal mineralization, including formation of porphyry copper deposits. Critical magma degassing parameters are the depth, chemistry and style of fluid release during magma ascent, notably the behaviour of chlorine, a key metal-transporting ligand. Currently, understanding is limited by restricted data on fluid-melt partitioning of chlorine as a function of pressure and magma chemistry, and the complex interplay between the two that occurs in polybaric magmatic systems. Here we present experimental determinations of chlorine partitioning as a function of fluid and melt composition at pressures from 50 to 800 MPa. We provide, for the first time, a quantitative understanding of chlorine and copper evolution that is valid for shallow, deep or transcrustal differentiation and degassing. Monte Carlo simulations using our new data reproduce the chemical evolution of melt inclusions from arc volcanoes and fluid inclusions from upper crustal intrusions and porphyry copper deposits. Our results not only provide a novel chemical framework for understanding magma degassing, but quantify the primacy of magmatic chlorine concentration at the point of fluid saturation in promoting efficient copper extraction from magmas.

## Abstract (web summary)

Chlorine behaviour during complex, polybaric arc magma degassing is poorly understood. Here, the authors show that chemical feedbacks during coeval magma differentiation and degassing account for the Cl record at both volcanoes and ore deposits, and quantify the role of Cl in efficient copper extraction during degassing.

## Introduction

Volatile-rich subduction zone magmas release substantial volumes of halogen-bearing and sulphur-bearing H_2_O–CO_2_ vapours, brines and supercritical fluids as they ascend and crystallize. Volcanic gas plumes and hydrothermal ore deposits are two manifestations of this degassing process. Although there is consensus that volatile-rich magmas are responsible for arc volcanism, plutonism and hydrothermal mineralization^1–3^, the manner of fluid release is keenly debated, with decompression (“first-boiling”) or cooling and crystallization (“second-boiling”) the main drivers^4^. Conventional concepts predicated on second-boiling of large, relatively volatile-poor (≤5 wt% H_2_O) melt-rich “magma chambers” in the shallow crust^5–7^ are now being challenged by models of vertically extensive, long-lived, mid- to lower-crustal crystal mushes containing a volatile-rich (8–15 wt% H_2_O) intergranular melt^1,8–13^. Such high dissolved H_2_O concentrations create problems for standard approaches to tracking magma degassing using H_2_O and CO_2_ because the latter is extensively degassed by the time magmas reach shallow sub-volcanic or ore-forming domains. More generally, emerging views on these “trans-crustal” magmatic systems^4,13,14^ invoke volatile release from chemically diverse arc melts over a wide pressure range that need to be considered in modelling both volcano degassing and hydrothermal ore formation.

**Fig. 1:**
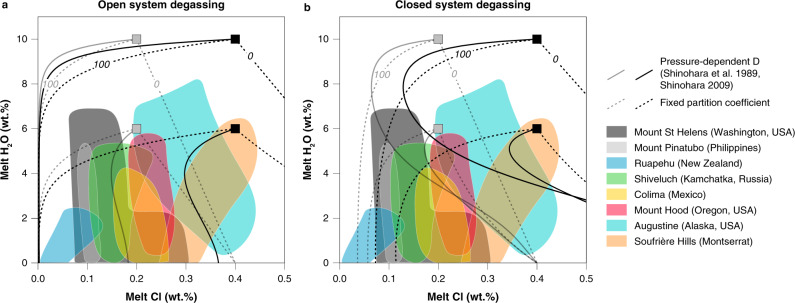
Chlorine degassing in arc volcanoes. Chlorine concentrations of rhyolitic melt inclusions (MI) from eight different arc volcanoes as a function of H_2_O concentrations (a proxy for pressure)^34,35,79–84^. Solid curves show, **a** open system^85^ and **b** closed system^86^ degassing trends calculated using the pressure-dependant $D_{{\mathrm{{Cl}}}}^{{\mathrm{{fluid}}}/{\mathrm{{melt}}}}$ calibration of refs. ^18,19^ for concurrent decompression and crystallization (assuming 50% crystallization along the degassing path) for four fluid-saturated magmas (6 and 10 wt% H_2_O, Cl concentrations of 2000 ppm (grey) and 4000 ppm (black). The strong mismatch between sub-vertical trends in the MI arrays and modelled degassing paths highlights the need for improved Cl partitioning data and a better understanding of feedbacks between degassing and crystallization.

First- and second-boiling processes impact differently on the behaviour of incompatible fluid-mobile elements, such as chlorine and copper, during degassing. In natural systems, fluid-mobile elements evolve along paths intermediate between these two end-members through competition between enrichment via crystallization and depletion by fluid exsolution (Fig. 1). The exact path followed depends on the initial volatile budget and competition (or feedback) between the two degassing regimes. The impact on melt and fluid compositions is modulated by the fluid-melt partition coefficients for the volatile species of interest at the point of exsolution. As copper uptake by fluids depends critically on fluid salinity^15–17^, so the behaviour of chlorine is key to elucidating both degassing style and ore formation. Only by quantifying chemical feedbacks across the full range of differentiation paths can we evaluate the magmatic cycling of volatiles and ore metals under various degassing scenarios. Furthermore, by using Cl and H_2_O as probes of the degassing process, we circumvent problems with traditional use of CO_2_ and H_2_O (e.g. ref. ^4^) that arise because extensively degassed, evolved magmas typically have very low dissolved CO_2_ concentrations.

**Fig. 2:**
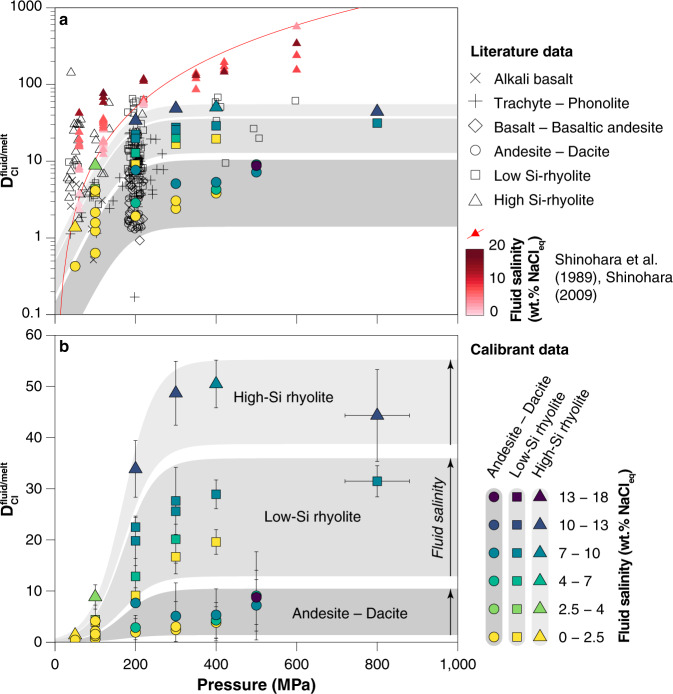
Experimental $D_{{\mathrm{{Cl}}}}^{{\mathrm{{fluid}}}/{\mathrm{{melt}}}}$ as a function of pressure. **a** Literature data for $D_{{\mathrm{{Cl}}}}^{{\mathrm{{fluid}}}/{\mathrm{{melt}}}}$ (black and red symbols) compared to calibrant dataset (coloured symbols). Ref. ^19^ calibration of $D_{{\mathrm{{Cl}}}}^{{\mathrm{{fluid}}}/{\mathrm{{melt}}}}$ as a function of pressure shown as red curve. Experimental data used to calibrate this model involve fluids of variable salinity, as shown by the red shading of the data points. Small displacements were added along the abscissa to better visualize the cluster of experiments at 200 MPa. **b** New experimental data, along with data from refs. ^17,30^ used to calibrate Eq. (1). Calibrant data are subdivided by melt composition (symbol shape) and fluid salinity (symbol colour). See references and data selection criteria in the “Methods” section.

Early experiments^18–20^ identified pressure as a key control on chlorine partition coefficients ($D_{{\mathrm{{Cl}}}}^{{\mathrm{{fluid}}}/{\mathrm{{melt}}}}$). However, melt inclusions in crystals from arc magmas show little change in chlorine concentration as degassing and crystallization proceed, contradicting predictions based on the available $D_{{\mathrm{{Cl}}}}^{{\mathrm{{fluid}}}/{\mathrm{{melt}}}}$ data^19^ (Fig. 1). Subsequent experiments^20–25^ highlighted the additional influences of major element composition of melts and total chlorine content of the system, yet many of these studies used melt compositions distinct from typical subduction zone magmas, hampering their quantitative application to arc volcanism and mineralization. Moreover, these studies often relied on imprecise mass balance methods (especially those with two-phase fluid assemblages) to determine fluid composition. Experiments utilizing robust mass-balance methods were very limited in pressure and composition space (Fig. 2a). Consequently, previous attempts to model Cl and Cu behaviour during degassing^26–28^ provided useful insights, but were unable to describe the complex chemical feedbacks inherent in the evolution of chemically diverse, polybaric magmatic systems.

## Results

### Experiments

To address the lack of appropriate fluid-melt partitioning data we performed fluid-saturated experiments at 50–800 MPa, 800–950 °C using as starting materials natural metaluminous calc-alkaline rhyolite, dacite and andesite to which were added NaCl–KCl–HCl solution and quartz cylinders. An in-situ quartz fracturing technique^17,29^ was used to trap fluid inclusions at run temperature enabling direct determination of $D_{{\mathrm{{Cl}}}}^{{\mathrm{{fluid}}}/{\mathrm{{melt}}}}$. Our experimental dataset (*n* = 19) was augmented by data from ref. ^17^ and a set of low-salinity (<20 wt% NaCl_eq_) fluid + andesite experiments^30^. Full experimental details are provided in the “Methods” section; run conditions and analyses in Supplementary Data. While Cl species in fluids in equilibrium with metaluminous calc-alkaline melts typically includes a mixture of NaCl, KCl, HCl, FeCl_2_ and trace metal chloride species (e.g., ${\mathrm{CuC}}^{-}_{2}, {\mathrm{ZnCl}}_{2}, {\mathrm{PbCl}}_{2}$, etc), we did not quantify the speciation of Cl in our experiments. Instead, we use the NaCl equivalent notation (NaCl_eq_) throughout this study to describe the total Cl concentration of the fluid, irrespective of its speciation.

For a given starting composition and fluid salinity we observe an increase in $D_{{\mathrm{{Cl}}}}^{{\mathrm{{fluid}}}/{\mathrm{{melt}}}}$ with pressure until 200–300 MPa whereupon $D_{{\mathrm{{Cl}}}}^{{\mathrm{{fluid}}}/{\mathrm{{melt}}}}$ begins to plateau, becoming relatively constant above 400 MPa (Fig. 2b; cf. ref. ^31^). This is in marked contrast to an existing $D_{{\mathrm{{Cl}}}}^{{\mathrm{{fluid}}}/{\mathrm{{melt}}}}$ calibration for silicic melts^19^ that suggests steady increase in $D_{{\mathrm{{Cl}}}}^{{\mathrm{{fluid}}}/{\mathrm{{melt}}}}$with increasing pressure (Fig. 2a—red line)^18,19^. The apparent partial molar volume of NaCl in aqueous fluids ($\bar V_{{\mathrm{{NaCl}}}}^{{\mathrm{{fluid}}}}$) shows a similarly steep rise and plateau over the same pressure range^31^ (Supplementary Fig. 1a). Comparison of our partitioning data ($D_{\mathrm{{{Cl}}}}^{{\mathrm{{fluid}}}/{\mathrm{{melt}}}}$) with calculated $\bar V_{{\mathrm{{NaCl}}}}^{{\mathrm{{fluid}}}}$ for the experimental conditions highlights the strong similarity in behaviour (Supplementary Fig. 1b), consistent with the interpretation that thermodynamic properties of NaCl in the fluid phase exert the strongest control on $D_{\mathrm{{{Cl}}}}^{{\mathrm{{fluid}}}/{\mathrm{{melt}}}}$ as a function of pressure^31^. Our dataset also highlights the marked increase of $D_{\mathrm{{{Cl}}}}^{{\mathrm{{fluid}}}/{\mathrm{{melt}}}}$ with increasing melt SiO_2_ concentration (Fig. 2b) during calc-alkaline differentiation, and the non-Henrian dependence of $D_{\mathrm{{{Cl}}}}^{{\mathrm{{fluid}}}/{\mathrm{{melt}}}}$ on fluid salinity, emphasizing the importance of chemical feedbacks during differentiation and degassing.

Our $D_{\mathrm{{{Cl}}}}^{{\mathrm{{fluid}}}/{\mathrm{{melt}}}}$ data can be described in terms of these three interlinked controls by a logistic function of pressure (*P* in MPa) and an exponential function of melt SiO_2_ (wt%) and fluid salinity (wt% NaCl_eq_)

1 $$D_{{\mathrm{{Cl}}}}^{\frac{{{\mathrm{{fluid}}}}}{{{\mathrm{{melt}}}}}} = D_0 \cdot \frac{{e^{a \cdot {\mathrm{{NaCl}}}_{{\mathrm{{fluid}}}} + b \cdot {\mathrm{{SiO}}}_{2_{{\mathrm{{melt}}}}}}}}{{1 + e^{\frac{{P_{\mathrm{{0}}} - P}}{{\mathrm{{c}}}}}}}$$

where *D*_0_, *a*, *b* and *c* are constants and *P*_0_ is the inflection point. A weighted regression of the data (*n* = 38) yields the following parameter values:

$$\left\{ {\begin{array}{*{20}{l}} {D_0 = 0.005654 \pm 0.005120\left( {1SE} \right)} \hfill \\ {a = 0.05749 \pm 0.01731} \hfill \\ {b = 0.1099 \pm 0.0128} \hfill \\ {P_0 = 172.8 \pm 10.7} \hfill \\ {c = 40.42 \pm 4.74} \hfill \end{array}} \right.$$

See the “Methods” section and Supplementary Figs. 2–4 for further details of the fitting procedure, quality of fit, and parameter dependencies. Application of this empirical parameterization of $D_{{\mathrm{{Cl}}}}^{{\mathrm{{fluid}}}/{\mathrm{{melt}}}}$allows for modelling the partitioning of Cl into magmatic derived fluids throughout differentiation and degassing of chemically diverse (from basaltic andesite to rhyolite) arc magmas for a wide range of possible scenarios. We emphasize that this parametrization can only be used for metaluminous calc-alkaline magma and does not reproduce experimental data from peraluminous or alkaline systems (Supplementary Figure S5).

### Modelling magma degassing

To explore chlorine evolution during magma degassing we incorporated Eq. (1) into a Monte Carlo simulation model that evaluates a range of degassing scenarios by randomly combining first-boiling (“1B”) and second-boiling (“2B”) degassing steps to define degassing paths for different starting pressures and different starting magma compositions along a metaluminous calc–alkaline differentiation series. As such, some paths are isobaric and others are mixtures of 1B and 2B steps (see the “Methods“ section). All modelled paths start at the point of fluid saturation (defined as a partial pressure, $P_{{\mathrm{{H}}}_2{\mathrm{{O}}}}$, or melt H_2_O concentration) and always falls along the defined metaluminous calc–alkaline compositional trend (Supplementary Fig. S6) with the onset of water saturation varying between 200 and 800 MPa at melt compositions between 57 and 70 wt% SiO_2_. After each 1B or 2B degassing step, the instantaneous fluid fraction was assumed to be in equilibrium with the melt. All modelled paths terminate when the melt has fully crystallized (see the “Methods” section for details). While all degassing paths start at a melt fraction of 1 at varying SiO_2_ concentrations, the evolution of the melt fraction and melt composition was parametrized from a compilation of experimental data (see the “Methods” secton). This range of fluid saturation conditions (both in pressure and melt composition) and variable degassing paths serves to examine a wide range of possible degassing scenarios from shallow, deep or trans-crustal degassing of andesitic to rhyolitic magmas. For each modelled path we compute also the weighted average of the fluid fractions degassed at each increment in order to approximate the aggregated fluid composition discharged from the entire underlying system^28^. Note that, in nature, degassing will be some mixture of instantaneous and aggregated degassing modes depending on the style of fluid ascent and accumulation throughout degassing. Our models implement the latest H_2_O–CO_2_ solubility algorithm^32^; alternative formulations do not greatly change the key findings. We do not explicitly include the effects of CO_2_ on fluid chemistry or chlorine partitioning, although we recognize that this is a rich area for further study, especially if arc magmas have high initial CO_2_ concentrations^33^.

## Discussion

### The fate of chlorine during magma degassing

**Fig. 3:**
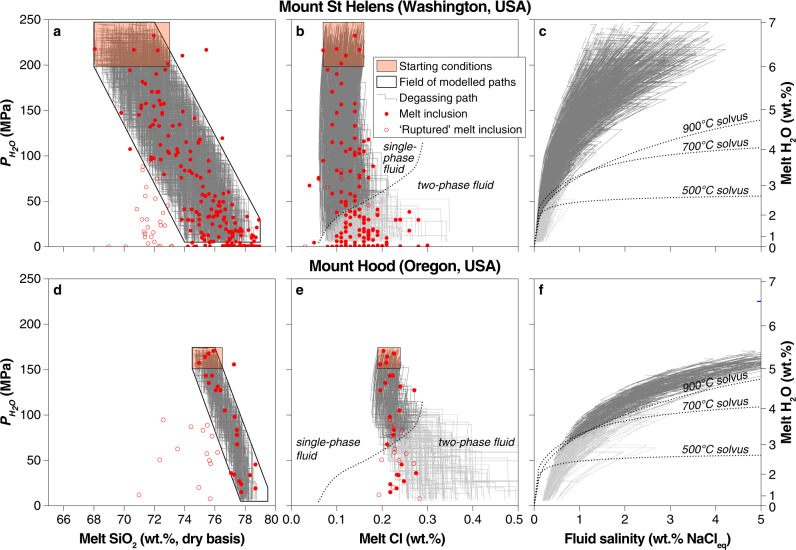
Monte Carlo degassing model applied to volcanic melt inclusions. **a** and **d** The SiO_2_ and H_2_O concentrations of the melt inclusions (MIs) from Mount St. Helens and Mount Hood^34,35^ were used to generate a subset of simulated degassing paths that start within the orange rectangle and encompass the full MI dataset for each volcano. **b** and **e** Chlorine concentration of MIs compared to that of of H_2_O-saturated melts predicted by our model. A strong correlation is observed for both systems until the vapour–brine solvus^56^ is intersected (greyed-out paths). **c** and **f** Salinity of the instantaneous fluid increments in equilibrium with the modelled silicate melt at each step. As in **b** and **e**, lighter grey region shows bulk fluid compositions that would experience vapour–brine separation.

We tested our approach against melt inclusion (MI) data from two arc volcanoes, Mount St. Helens^34^ and Mount Hood^35^, by identifying a subset of random differentiation paths that matches the observed range of SiO_2_ and $P_{{\mathrm{{H}}}_2{\mathrm{{O}}}}$ (Fig. 3a, d). We tuned the initial chlorine concentration in the melt to match that of the least differentiated MIs from both volcanoes in order to model the chlorine concentration of fluid and melt throughout ascent, crystallization and degassing (Fig. 3b, e). Our model reproduces faithfully the chlorine concentration of the evolving melt (Fig. 3b, e) as well as providing compositions of the coexisting fluids (Fig. 3c, f). Excursions of modelled melt compositions only occur after intersecting the NaCl–H_2_O solvus at low pressure when the single-phase fluid separates into a hypersaline liquid (or ‘brine’) and a low salinity vapour (Fig. 3), a process not accounted for by Eq. (1). By reproducing the near-constant chlorine contents of MIs with widely variable H_2_O contents, our model results emphasize the chemical feedbacks imposed on $D_{{\mathrm{{Cl}}}}^{{\mathrm{{fluid}}}/{\mathrm{{melt}}}}$ by decompression (decreasing H_2_O solubility), crystallization (increasing SiO_2_), and evolving fluid salinity during degassing in a manner not possible using existing parameterizations (Fig. 1). The ability to reproduce volcanic melt inclusion data highlights how our parametrization of $D_{{\mathrm{{Cl}}}}^{{\mathrm{{fluid}}}/{\mathrm{{melt}}}}$ can be used to successfully predict the compositions of magmatic fluids released during differentiation and degassing even in complex crystallization and decompression scenario^13^.

**Fig. 4:**
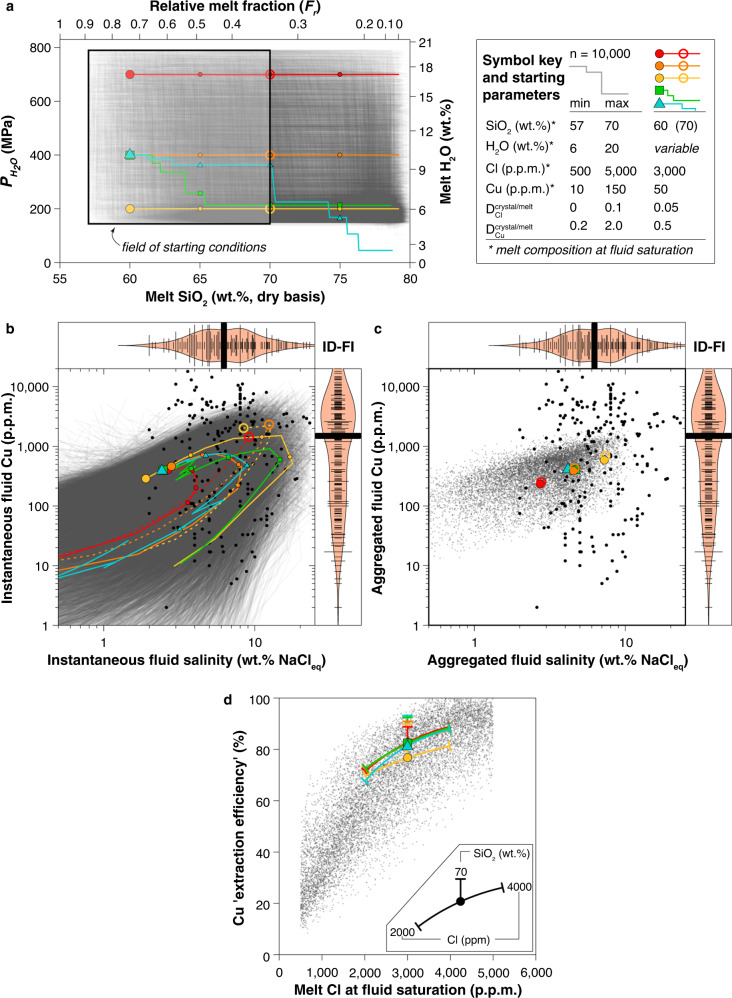
Monte Carlo simulations of degassing paths. **a** 10,000 input random degassing and crystallization paths. Degassing begins at fluid saturation for a specified melt H_2_O concentration or $P_{\mathrm{{{H}}}_2{\mathrm{{O}}}}$ (vertical axis). In the case of H_2_O-saturated melts, $P_{\mathrm{{{H}}}_2{\mathrm{{O}}}}$ corresponds to lithostatic pressure (*P*_lith_); if melts also contain CO_2_, then $P_{\mathrm{{{H}}}_2{\mathrm{{O}}}}$ < *P*_lith_. Horizontal axes denote melt fraction (top) and melt SiO_2_ concentration (bottom). Prior to onset of fluid saturation, H_2_O, Cl and Cu concentrations in the melt evolve from their initial values through crystallization. Five reference degassing paths differing only in H_2_O content at the onset of fluid release are highlighted in colour to represent plausible scenarios for magmas differentiating isobarically (2B paths at 700, 400, and 200 MPa) or during decompression (mixed 1B–2B paths originating at 400 MPa). Large coloured symbols denote the beginning of the reference paths; smaller coloured symbols on each path are shown at 65, 70 and 75 wt% SiO_2_. The starting parameters for the reference paths and the corresponding range for all simulated paths are indicated in the inset. Salinity and Cu concentration of the instantaneous fluid (**b**) produced at each increment along the degassing path (large and small coloured symbols on each path as in **a**) and of the aggregated fluid (**c**) for each path, compared to the composition of intermediate-density fluid inclusions (ID-FI) from intrusive systems (black points; data from ref. ^38^). Bean plots display the density distribution and the median (thick black lines) of ID-FI compositions from intrusive systems. **d** Copper extraction efficiency plotted as a function of the melt Cl concentration at fluid saturation. The impact of varying SiO_2_ or Cl concentration of the melt at fluid saturation is shown for the five coloured reference paths.

To explore magma degassing more broadly, we modelled the composition of the silicate melt and fluid (instantaneous and aggregated) for incremental equilibrium degassing using 10,000 mixed 1B–2B degassing scenarios (Fig. 4a; Supplementary Figs. 7–11). The minimum pressure was set to 150 MPa to avoid crossing the NaCl–H_2_O solvus. Two-phase (brine + vapour) fluid degassing will occur in some very shallow (<150 MPa) Cl-rich systems. Nonetheless, limited brine partitioning data are also available in the Supplementary Data for use in brine/vapour/melt modelling beyond the limits of the models presented here. Within this larger dataset of paths, several example paths are highlighted in Fig. 4a to represent common or plausible scenarios for natural magmatic systems. These paths are meant to represent (1) shallow crystallization-driven degassing (2B-second boiling) of evolved (yellow dashed) or intermediate (yellow) magma reservoirs. (2) Similar 2B degassing from deeper (400 or 800 MPa) much more hydrous intermediate to evolved magmas (orange and red) and (3) complex decompression-driven and crystallization-driven degassing paths leading to volcanic eruption (blue) or pluton emplacement (green). The yellow paths were chosen to mimic the “standard” model for porphyry ore deposit formation, whereby hydrous andesitic to rhyolitic magmas degas by second-boiling in large, epizonal plutons at ~200 MPa^5,6^. The deeper more hydrous degassing paths are designed to mimic the conditions where deep compressional magma degassing might occur^9,11^.

Our results show that salinity of aggregated fluids is controlled primarily by melt chlorine and H_2_O concentrations at the onset of fluid saturation (Supplementary Fig. 7). The subsequent degassing path has negligible influence on aggregated fluid salinity (Fig. 4c; Supplementary Fig. 8), but important differences exist between instantaneous fluid salinities produced by 1B and 2B degassing increments (Fig. 4b). In detail, 1B steps show a decrease in both melt chlorine concentration and fluid salinity, whereas 2B steps typically show a modest increase in both parameters due to the competing effects of chlorine extraction into the fluid and enrichment in the melt through coeval crystallization. For nearly all modelled paths, fluid salinity first increases then decreases as magmas ascend and differentiate (Fig. 4b; Supplementary Figs. 9, 10), producing a marked peak in salinity for instantaneous fluids released from moderately evolved melts (65–75 wt% SiO_2_). The example isobaric paths starting with more evolved melts show that they do initially exsolve higher salinity fluids (Fig. 4b—dashed paths) compared to fluids exsolved from more intermediate melts (solid paths). However, the higher $D_{{\mathrm{{Cl}}}}^{{\mathrm{{fluid}}}/{\mathrm{{melt}}}}$ more rapidly depletes the melt of Cl and the overall fluid salinity drops very quickly throughout the remainder of degassing. As a result, these variations in the instantaneous fluid salinity due to melt evolution during degassing generally average out for the aggregated fluid.

Intermediate density (ID), supercritical fluid inclusions from magmatic rocks likely sample single-phase fluids released at different stages of magmatic differentiation^36^. This is supported by the observation that the salinity distribution of our modelled instantaneous fluids reproduces the salinity range of ID fluids recorded in fluid inclusions from barren plutons and porphyry ore deposits (Fig. 4b—black dots and bean plot)^28,37,38^. In terms of aggregated fluids, the median salinity (6 wt% NaCl_eq_) of ID fluid inclusions is best matched by an initial Cl/H_2_O weight ratio of ~0.04 (e.g. 2000 ppm Cl and 5 wt% H_2_O or 4000 ppm Cl and 10 wt% H_2_O; Fig. 4c; Supplementary Fig. 7).

### Implications for porphyry copper deposit formation

We calculated the copper concentration in both instantaneous and aggregated fluids and the extraction efficiency of copper by the fluid (i.e. mass ratio of copper in aggregated fluid to that in parental melt) using a salinity-dependant $D_{{\mathrm{{Cl}}}}^{{\mathrm{{fluid}}}/{\mathrm{{melt}}}}$ expression^17^. Experimental copper partitioning data for andesites^39^ show that this expression holds for the range of melt compositions modelled.

Modelled instantaneous fluids describe curved, clockwise trajectories in Cu-salinity space (Fig. 4b) with a peak in instantaneous fluid copper concentration after ~60–80% of fluid has been released (Supplementary Fig. 10). This peak corresponds to somewhat less silicic melt (60–75 wt% SiO_2_) than the peak in salinity (Fig. 4b; Supplementary Fig. 9). Melts that begin degassing at a later stage of differentiation (dashed paths Fig. 4; Supplementary Fig. 9) also achieve relatively high initial Cu concentrations in the fluid, due to higher salinity, but the Cu concentration of the fluids likewise drops quickly in a manner similar to the fluid salinity. This decoupling in the behaviour of copper and chlorine is due to the relatively higher fluid/melt partition coefficient for copper compared to chlorine at ID fluid salinities^17^. In terms of degassing style, melt copper concentrations drop with both 2B and, more markedly, 1B steps (Supplementary Fig. 8).

The overall range in copper concentrations in modelled aggregated fluids (Fig. 4c) is considerably less than for instantaneous fluids (Fig. 4b). Notably, the range of copper concentrations in instantaneous fluids reproduces most of the range of copper concentrations of ID fluid inclusions from natural systems^38,40^. The extreme of the range (5000–20,000 ppm Cu) cannot be reproduced by our model, suggesting that post-entrapment copper diffusion affected some ID fluid inclusions, especially at salinities <8 wt% NaCl_eq_^41,42^. Indeed, our calculated aggregated fluids from differentiation paths that yield the highest ID fluid salinities (~7 to 12 wt%) have copper concentrations below ~1500 ppm (Fig. 4c). Based on the results in Fig. 4, we suggest that, whereas porphyry copper deposits may be the product of the aggregated fluids discharge from a large magmatic system, ID fluid inclusions themselves represent discrete aliquots of fluid released from different portions of the system.

From a chemical perspective, the formation of economic porphyry copper mineralization requires both significant tonnage of Cu, which benefits from successful extraction from a large magmatic system^5,6,26,28^, as well as significant Cu grades that depend at least in part upon relatively high Cu concentrations in the fluid^38^. However, across all differentiation paths, the single strongest chemical control on Cu extraction efficiency is the chlorine concentration in the melt at the onset of fluid saturation (Fig. 4d). For example, increasing melt chlorine concentration from 500 to 3000 ppm imparts an almost eight-fold increase in copper extraction efficiency (Fig. 4d); minor improvement (5–10% relative) in extraction efficiency occurs where melts are more SiO_2_-rich at the onset of fluid saturation (Fig. 4d). Our example paths highlight that this feature is present for degassing from shallow or deep magmas and minimally affected by the mode of degassing. More evolved melts have slightly higher extraction efficiencies (Fig. 4d) but these melts on average have lower Cu concentrations available for extraction^43,44^. The possible presence of reduced sulphur in the system may promote saturation of sulphide melts or solids that can inhibit copper enrichment in residual melts by effectively increasing the bulk $D_{{\mathrm{{Cu}}}}^{{\mathrm{{crystal}}}/{\mathrm{{melt}}}}$. Although this reduces the copper available for extraction, the effect is trivial compared to the overarching influence of initial chlorine (Fig. 4d; Supplementary Fig. 11).

Our results demonstrate that porphyry copper mineralization is favoured under conditions where both copper and chlorine become enriched in residual melts prior to fluid saturation. Copper-rich magmas are scarce on Earth^43,44^ making it difficult to ascribe ore formation simply to very high initial copper concentrations. Instead, because of its strong influence on extraction efficiency, magmatic chlorine exerts much greater leverage on porphyry copper mineralization than magmatic copper. In continental arcs, melt Cu concentration invariably peaks at ~100–200 ppm around 55 wt% SiO_2_ during differentiation^44^, then slowly declines to values between ~50–10 ppm from 60 to 70 wt% SiO_2_. This behaviour, due to the onset of sulphide saturation, has been incorporated into our simulations through variations in $D_{{\mathrm{{Cu}}}}^{{\mathrm{{crystal}}}/{\mathrm{{melt}}}}$ that account for possible sulphide precipitation during degassing (see the “Methods” section for details).

In contrast to copper, chlorine melt concentrations increase throughout differentiation. Early onset of fluid saturation (basaltic to andesitic degassing) liberates fluids at low $D_{\mathrm{{{Cl}}}}^{{\mathrm{{fluid}}}/{\mathrm{{melt}}}}$(Fig. 2) and before the chlorine concentration of the melt has been enriched significantly through crystallization, resulting in low fluid salinity (Supplementary Fig. 9b) and low copper extraction efficiency (Fig. 4d). Therefore, our model suggests that optimal conditions for copper extraction are met for the case of intra-crustal crystallization-driven degassing (second boiling) of Cl-rich, hydrous derivative dacitic to rhyolitic (65–70 wt% SiO_2_) magmas, whereby elevated pressure keeps H_2_O in solution enabling sufficient chlorine and SiO_2_ enrichment for high $D_{\mathrm{{{Cl}}}}^{{\mathrm{{fluid}}}/{\mathrm{{melt}}}}$ and thus high $D_{\mathrm{{{Cl}}}}^{{\mathrm{{fluid}}}/{\mathrm{{melt}}}}$. Subsequent decompression, crystallization and degassing of derivative dacitic to rhyolitic (65–70 wt% SiO_2_) magmas at mid-crustal pressures (400–200 MPa) results in efficient extraction (high $D_{{\mathrm{{Cu}}}}^{{\mathrm{{fluid}}}/{\mathrm{{melt}}}}$) of copper into the fluid (Fig. 4d).

### Linking arc volcanoes and hydrothermal ore deposits

We present a chemical framework to evaluate chlorine–water degassing systematics in transcrustal, subduction-related magmatic systems. Through Monte Carlo models of polybaric, chemical differentiation of fluid-saturated magmas we quantify the strong chemical feedbacks between degassing pressure and the chemistry of coexisting melt and fluids that helps bridge the “missing link”^19^ between degassing behaviour of volcanoes and formation of hydrothermal ore deposits. Moreover, the use of H_2_O and Cl as tracers of magmatic degassing provides a companion to more conventional pairs, such as H_2_O and CO_2_, when examining water-rich systems. Significantly, we show how a combination of elevated magmatic chlorine and moderate to deep differentiation of H_2_O-rich magmas^45^ are required to optimize efficient copper extraction upon magma emplacement in the upper to middle crust.

The primacy of magmatic chlorine in controlling volcanic fluid properties and copper extraction efficiency emphasizes the need to understand chlorine cycling through subduction zones. The global dataset for arc basalts (48–50 wt% SiO_2_) shows considerably greater variability in concentrations of chlorine (~50 to ≥5000 ppm)^46^ than of H_2_O (1.3–7.1 wt%)^47^. At any subduction zone the ultimate source of chlorine is likely subducted oceanic crust and sediments^48–50^. Variations in supply of chlorine (and other halogens) along an individual arc segment may be related to subducted fracture zones or seamounts^49^, interaction with chlorine-rich crustal lithologies, or sequential enrichment via cyclic differentiation^9^. The chemical controls and feedbacks on chlorine degassing and copper extraction efficiency identified here work in tandem with other physical controls, including geodynamic stress regime and fluid focussing, to influence the size and location of porphyry copper deposits. Thus, even chlorine-rich volcanic systems (e.g. Augustine, Soufrière Hills, Fig. 1) require additional physical conditions to yield a fluid exsolution history primed for efficient copper extraction and high-grade mineralization. Nonetheless, magmatic chlorine concentrations, retrieved from melt inclusions or from chlorine-bearing minerals in porphyry systems (e.g. apatite, amphibole, biotite)^51^, represent novel tools for modelling magma evolution and evaluating copper prospectivity.

## Methods

### Experiments

Experiments were performed in Inconel^®^ and TZM-alloy cold-seal hydrothermal pressure vessels (CSPV) and piston cylinder apparatus at the University of Bristol. For all experiments, ~50 wt% powdered natural igneous starting material was mixed with ~50 wt% aqueous NaCl–KCl–HCl solution (in Cl proportions 20:10:1) and was loaded into welded 3.8 mm (CSPV) or 3 mm (piston cylinder) OD gold capsules. Samples of rhyolite, dacite, and andesite from Laguna del Maule, Chile were used for the experiments (samples held as part of the collection at the University of Bristol). Compositions of the starting materials can be found in Supplementary data file.

Experiments at 100–400 MPa were performed in custom-machined Inconel713LC CSPVs of 12″ length and ID/OD = 0.25″/1.0″ (ƒO_2_ ~ NNO + 0.6). Using a rod-centreing, double-cone sealing technique, these vessels performed successfully for more than 1000 h total run-time at 800–850 °C (Type K thermocouples ±5 °C) at 300 and 400 MPa following more than 5000 h run-time at lower pressures. Run pressure was measured using both a Wika HP-2-S digital pressure transducer (0–420 MPa working range ±2 MPa uncertainty) and a certified analogue Heise pressure gauge (0–700 MPa working range ±10 MPa uncertainty). In experiments performed with the Inconel713LC CSPV apparatus (ƒO_2_ ~ NNO + 0.6), a cylindrical 2.5 mm OD core of unfractured, inclusion-free Brazilian quartz was loaded into the capsule to trap fluid inclusions. After 5 days at elevated pressure and temperature (i.e. when fluid and melt had equilibrated) the quartz was fractured in situ^17,29^ using a magnetic filler rod control system. Following fracturing, the experiments were left to run another 3 days to allow the quartz to “heal” and trap fluid inclusions. At the end of the experiment, a modest quench rate was used to cool the capsule to room temperature over ~1 min, which prevents re-fracturing the quartz while still allowing for the transformation of the melt to homogeneous glass.

Andesitic-dacitic experiments (CMPA141 and CPMA281) were run in a TZM-alloy Ar–H_2_ pressurized (ƒO_2_ ~ NNO) CSPV to achieve the higher temperatures (950 °C) necessary to maintain significant melt fraction. Approximately 20–30% crystallization of the melt in these runs resulted in a trachydacitic final melt composition. The lower bulk SiO_2_ concentration meant that melt was not close to silica saturation, thus quartz cores could not be used to trap fluid inclusions; for these runs mass balance calculations were used to determine Cl partition coefficients. Type K thermocouples and digital pressure transducers were used to control run pressure (±2 MPa) and temperature (±5 °C) similar to CSPV runs.

Experiments at 800 MPa were run in half-inch (12.7 mm) end-loaded piston cylinder apparatus using a salt-Pyrex assembly. Gold capsules (3 mm OD; 10 mm long) were filled with the same glass starting materials and NaCl–KCl–HCl aqueous solution, along with a pre-fractured quartz core (in-situ fracturing is not yet possible in piston cylinder experiments at these conditions). ƒO_2_ control was established by placing a small (2 mm OD; 3 mm long) Ni–NiO–H_2_O-filled gold capsule at the bottom of the sample capsule. Temperature was measured with a Pt–Rh thermocouple in contact with the capsule. A friction correction appropriate to the salt-Pyrex assembly^52^ was applied. Formation of CO_2_ due to infiltration of elemental carbon from the graphite furnace was minimized by coating the capsules with haematite prior to placing them in the assembly. Examination of run-product fluid inclusions, prior to microthermometry, using a Thermo Scientific DXRxi Raman spectrometer at 532 nm using variable laser power and scan times revealed no detectable CO_2_ in any inclusions.

### Analysis

Compositions of the silicate glasses and salinity of the fluid inclusions recovered after quenching were measured directly by electron probe microanalysis (EPMA) and microthermometry, respectively. Solid run products were mounted in one-inch epoxy rounds, polished and carbon-coated. Major oxides (SiO_2,_ Al_2_O_3_, TiO_2_, MnO, MgO, FeO, CaO, K_2_O, Na_2_O, P_2_O_5_) and volatiles (S, Cl, F) in glasses were acquired at University of Bristol using a five spectrometer JEOL JXA8530F Hyperprobe. An accelerating voltage of 15 kV and a defocussed beam diameter of 10 µm was used for all glass analyses. Major oxides were measured at a beam current of 2 nA to minimize diffusive loss of Na from the analytical volume^53^; S, Cl, F were measured subsequently on the same spot using 20 nA. Analytical accuracy was assessed by measuring a hydrous rhyolitic glass secondary standard. Between 12 and 20 analyses were averaged for each experiment. Water concentrations were determined using the volatiles by-difference (VBD) method. Average glass analyses for all experimental runs are reported in Supplementary Data. Experimental melts at 800 MPa had unquenchable water contents (~20 wt%) resulting in small (~1 µm) quench bubbles across the experimental glass. Strong disequilibrium Cl mobility during this process would result in a heterogeneous run-product glass, which is not observed in 800 MPa experiments. In the end-member case where Cl is able to equilibrate during the formation of quench bubbles no difference between the measured Cl concentrations of the melt ($\left[ {{\mathrm{{Cl}}}} \right]_{{\mathrm{{melt}}}}^{{\mathrm{{measured}}}}$) and the equilibrium value ($\left[ {{\mathrm{{Cl}}}} \right]_{{\mathrm{{melt}}}}^{{\mathrm{{equilibrium}}}}$) is expected. In the other end-member case where pure water is exsolved during quench (slow Cl diffusion) the melt will lose ~10% water, resulting in an 11% (relative) increase in the measured Cl concentration $\left[ {{\mathrm{{Cl}}}} \right]_{{\mathrm{{melt}}}}^{{\mathrm{{measured}}}}$. Without any way to confirm the salinity of the quench bubbles we have chosen to average $\left[ {\mathrm{{{Cl}}}} \right]_{\mathrm{{{melt}}}}^{\mathrm{{{measured}}}}$ and 0.9*$\left[ {\mathrm{{{Cl}}}} \right]_{{\mathrm{{melt}}}}^{{\mathrm{{measured}}}}$, as the true value for $\left[ {{\mathrm{{Cl}}}} \right]_{{\mathrm{{melt}}}}^{{\mathrm{{equilibrium}}}}$ lies between these extremes. An additional ±5% relative uncertainty is added to account for uncertainty associated with quench bubbles. Bulk composition and Cl concentration of the silicate melt from each run can be found in Supplementary Data.

Microthermometric measurements were made using a Linkam THMS600 heating–freezing stage at University of Bristol. Measurements of the ice melting point (±0.1 °C) were input into the SALTY salinity model^54^ in order to convert ice melting temperatures into MVP salinity (NaCl_eq_). Starting material ratios of NaCl/KCl = 2/1 were assumed for the calculations; little impact is expected due to modification of this ratio as their colligative impacts on ice melting are indistinguishable^55^. During interaction with the melt some FeCl_2_ may have dissolved into the fluids; the presence of FeCl_2_ (2/3 of ions are Cl) instead of NaCl/KCl (1/2 of ions are Cl) results in a 33% increase in Cl concentration for the same $T_{{\mathrm{{melt}}}}^{{\mathrm{{ice}}}}$. However, the limited FeCl_2_ content predicted for our inclusions (<10% of the total solute)^17^ results in ≤3% underestimation of the Cl concentration, below the analytical scatter in the fluid inclusions assemblages.

At least fifteen synthetic fluid inclusion analyses were conducted from at least five assemblages to ensure a robust sampling of trapped fluids. Three of the new experiments produced co-existing vapour-brine assemblages. Brine salinity was evaluated using the halite and sylvite dissolution temperatures^54^. However, our $D_{{\mathrm{{Cl}}}}^{\mathrm{{{fluid}}}/{\mathrm{{melt}}}}$ parameterization and modelling focus on degassing of supercritical fluids and low to intermediate salinity vapours, not two-phase vapour–brine degassing. Nonetheless, measured salinity and partitioning behaviour for all experimental fluid(s) is provided in Supplementary Data.

### Literature data

Data from ref. ^30^ were used in the calibrant dataset to supplement the low SiO_2_ end of the melt evolution spectrum. The relevant experiments at 50 and 100 MPa were adjusted by applying the calculated^56^ brine–saturated vapour salinity value rather than using the reported bulk salinity calculated by mass balance. The adjusted vapour salinity was used to recalculate $D_{{\mathrm{{Cl}}}}^{\mathrm{{{fluid}}}/{\mathrm{{melt}}}}$ for those runs.

Additional literature datasets^18,20,22–25,57–67^ for $D_{{\mathrm{{Cl}}}}^{\mathrm{{{fluid}}}/{\mathrm{{melt}}}}$ presented in Fig. 2a were screened according to several criteria. Experiments in the two-phase field (vapour + brine) that used mass balance methods to calculate bulk fluid salinity were not included, as this technique is thermodynamically invalid. In a melt–vapour–brine system the Cl concentration of all three phases is invariant for a given melt composition, pressure and temperature. The non-ideal behaviour of Cl as a function of salinity and mismatch for the appropriate Cl in the melt means that calculations of $D_{{\mathrm{{Cl}}}}^{\mathrm{{{fluid}}}/{\mathrm{{melt}}}}$ for a “bulk salinity” ignore this behaviour and thus have limited utility. In addition, several slightly different formulations for $D_{{\mathrm{{Cl}}}}^{\mathrm{{{fluid}}}/{\mathrm{{melt}}}}$ seem to have been used in many of these studies. In order to standardize the data, the same calculation procedure for $D_{{\mathrm{{Cl}}}}^{\mathrm{{{fluid}}}/{\mathrm{{melt}}}}$ as that used in our new experiments has been applied to all raw fluid and melt Cl concentrations from each dataset to yield the values in Fig. 2a. Original published values and standardized $D_{{\mathrm{{Cl}}}}^{\mathrm{{{fluid}}}/{\mathrm{{melt}}}}$ calculations are available in the Supplementary Data along with other available melt–fluid parameters. Finally, we plotted in Fig. 2 only those runs that are relevant to the salinity range of most natural intermediate-density fluids (<20 wt.% NaCl_eq_).

### Parameterization of $D_{{\mathrm{{Cl}}}}^{\mathrm{{{fluid}}}/{\mathrm{{melt}}}}$

Parameters of Eq. (1) were determined by weighted non-linear regression (weighted by the reciprocal variance) using the calibrant dataset (Supplementary Fig. 2). The regression yielded a residual standard error of 2.603 on 32 degrees of freedom. Errors on the three predictors (i.e., $\sigma _{{\mathrm{{NaCl}}}_{{\mathrm{{fluid}}}}}$, $\sigma _{{\mathrm{{SiO}}}_{2_{{\mathrm{{melt}}}}}}$, *σ*_P_) were propagated onto the predicted $D_{{\mathrm{{Cl}}}}^{\mathrm{{{fluid}}}/{\mathrm{{melt}}}}$ via a first-order Taylor expansion^68^. Using this calibration, we can reproduce literature $D_{{\mathrm{{Cl}}}}^{\mathrm{{{fluid}}}/{\mathrm{{melt}}}}$ values for melt compositions similar to those used for the calibration (Supplementary Fig. 5). Literature data with alkaline, albitic or haplogranitic melt compositions show systematically higher values for $D_{{\mathrm{{Cl}}}}^{\mathrm{{{fluid}}}/{\mathrm{{melt}}}}$ than predicted by our model (Supplementary Fig. 5), likely due to the effects of varying melt structure on chlorine activity, mass balance errors, or the effects of unconstrained Cl reservoirs (e.g. apatite crystals) in the experiments that affect the mass balance calculations used to obtain fluid salinity.

### Modelling strategy

10,000 points defined by their melt H_2_O, SiO_2_, Cl and Cu concentrations were randomly drawn and considered to represent the degassing conditions of a wide range of plausible water-saturated metaluminous calc–alkaline magmas. Degassing paths starting from these points were generated by a random combination of first boiling (decreasing H_2_O) and second boiling (increasing SiO_2_). Within the population of random paths, specific degassing paths linked to common models for differentiation are highlighted for reference. Melt H_2_O concentrations of water-saturated magmas was linked to pressure through a parametrized equation (Supplementary Fig. 12). Similarly, melt fraction was aliased to melt SiO_2_ concentration (Supplementary Fig. 6). The relationship between melt fraction and SiO_2_ is a proportional one where *F* is set to 1 at the onset of fluid saturation regardless of the SiO_2_ concentration at the onset of degassing. The Cl and Cu composition of the remaining melt and the exsolved fluid were calculated after each degassing step using partitioning equations and mass conservation.

### Generation of random degassing paths

Considering a fluid-saturated magma (i.e., a system of melt + crystals + fluid), the melt mass fraction (*F*) is defined as

2 $$F = \frac{{m_{{\mathrm{{melt}}}}}}{{m_{{\mathrm{{melt}}}} + m_{{\mathrm{{crystals}}}} + m_{{\mathrm{{fluid}}}}}}$$

where *m* refers to mass. In the course of crustal magmatic evolution, *F* decreases as a result of crystallization (i.e., extraction of both crystals and fluid from the melt; *B*^c^ = 1) and/or decompression (i.e., extraction of fluid from the melt; *B*^d^ = 1). At each calculation step (i), we model this by randomly drawing values of 0 or 1 for $B_i^{\mathrm{{c}}}$ and $B_i^{\mathrm{{d}}}$. In cases where both decompression and crystallization occur, the algorithm favours the status quo and reprises the previous step. We found that this choice of “inertia” better reproduces the path of natural magmas that pond and differentiate at discrete depths and decompress near-adiabatically:

3 $$\left\{ {\begin{array}{*{20}{c}} {B_i^{\mathrm{{d}}} = 0\,{\mathrm{{or}}}\,1} \\ {B_i^{\mathrm{{c}}} = 0\,{\mathrm{{or}}}\,1} \end{array}} \right.\,{\mathrm{{then}}}\,\left\{ {\begin{array}{*{20}{c}} {{\mathrm{{if}}}\,B_i^{\mathrm{{d}}} \,\ne\, B_i^{\mathrm{{c}}},\,B_i^{\mathrm{{d}}} = B_i^{\mathrm{{d}}}\,{\mathrm{{and}}}\,B_i^{\mathrm{{c}}} = B_i^{\mathrm{{c}}}} \\ {{\mathrm{{if}}}\,B_i^{\mathrm{{d}}} = B_i^{\mathrm{{c}}},\,B_i^{\mathrm{{d}}} = B_{i - 1}^{\mathrm{{d}}}\,{\mathrm{{and}}}\,B_i^{\mathrm{{c}}} = B_{i - 1}^{\mathrm{{c}}}} \end{array}} \right.$$

Let *δ*_F_ be the variation in melt fraction (F) between two successive steps. In order to extract a comparable mass of fluid from decompression or crystallization steps, the ratio (*r*_*δ*_) between the size (mass fraction of degassed H_2_O) of a crystallization step ($\delta _{\mathrm{{F}}} = \delta _i^{\mathrm{{c}}}$) and a decompression step ($\delta _{\mathrm{{F}}} = \delta _i^{\mathrm{{d}}}$) was varied randomly between 5 and 15:

4 $$\delta _i^c = r_\delta \times \delta _i^d$$

and $\delta _i^d$ was varied between 0.005 and 0.015. Considering an initial system of 100% of fluid-saturated melt, conservation of mass dictates that, at every step (*i*), the mass of each component is given by

5 $$\left\{ {\begin{array}{*{20}{c}} {\begin{array}{*{20}{c}} {m_{{\mathrm{{melt}}}_i} = m_{{\mathrm{{melt}}}_{i - 1}} - \delta _F \cdot m_{{\mathrm{{melt}}}_0} = m_{{\mathrm{{melt}}}_0} \cdot F_i} \hfill\\ {m_{{\mathrm{{crystals}}}_i} = m_{{\mathrm{{crystals}}}_{i - 1}} + B_i^{\mathrm{{c}}} \cdot \delta _i^{\mathrm{{c}}} \cdot \left( {1 - {\mathrm{{H}}}_2{\mathrm{{O}}}_{{\mathrm{{sat}}}_i}} \right) \cdot m_{{\mathrm{{melt}}}_0}} \hfill\\ {m_{{\mathrm{{water}}}_i} = m_{{\mathrm{{water}}}_{i - 1}} + B_i^{\mathrm{{c}}} \cdot \delta _i^{\mathrm{{c}}} \cdot {\mathrm{{H}}}_2{\mathrm{{O}}}_{{\mathrm{{sat}}}_i} \cdot m_{{\mathrm{{melt}}}_0} + B_i^{\mathrm{{d}}} \cdot \delta _i^{\mathrm{{d}}} \cdot m_{{\mathrm{{melt}}}_0}} \end{array}} \end{array}} \right.$$

with $H_2O_{sat_i}$ being the mass fraction of water dissolved in the melt:

6a $${\mathrm{{H}}}_2{\mathrm{{O}}}_{{\mathrm{{sat}}}_i} = B_i^{\mathrm{{d}}} \cdot \frac{{{\mathrm{{H}}}_2{\mathrm{{O}}}_{{\mathrm{{sat}}}_{i - 1}} \cdot F_{i - 1} - \delta _i^{\mathrm{{d}}}}}{{F_i}} + B_i^{\mathrm{{c}}} \cdot {\mathrm{{H}}}_2{\mathrm{{O}}}_{{\mathrm{{sat}}}_{i - 1}}$$

6b $$= {\mathrm{{H}}}_2{\mathrm{{O}}}_{{\mathrm{{sat}}}_{i - 1}} - B_i^{\mathrm{{d}}} \cdot \frac{{\delta _i^{\mathrm{{d}}}}}{{F_i}} \cdot \left( {1 - {\mathrm{{H}}}_2{\mathrm{{O}}}_{{\mathrm{{sat}}}_{i - 1}}} \right)$$

### Pressure estimates

Water solubility was calculated at 800 °C from 0 to 900 MPa for four experimental melt compositions ranging from andesite to rhyolite using MagmaSat^32^ (Supplementary Fig. 12 and Supplementary Data). At each step of the model the partial pressure of H_2_O in the melt ($P_{{\mathrm{{H}}}_2{\mathrm{{O}}}}$) and was estimated following the equation:

7 $$P_{{\mathrm{{H}}}_2{\mathrm{{O}}}_i}\left( {{\mathrm{{MPa}}}} \right) = \frac{{1430.5}}{{1 + 0.052637 \cdot \left( {{\mathrm{{H}}}_2{\mathrm{{O}}}_{{\mathrm{{sat}}}_i}} \right)^{ - 1.6958}}}$$

In the range of pressure and composition relevant for the present study (100–800 MPa, andesite to rhyolite), the pressure estimated using this equation lies within 5 rel.% of that calculated with MagmaSat (grey field in Supplementary Fig. 12).

### SiO_2_ vs. melt fraction

The relative mass fraction of melt (*F*_r_, in the system melt + crystals) was defined as a function of SiO_2_ (in wt%; anhydrous basis) along a calc-alkaline differentiation trend by parameterizing published water-saturated experimental data^69–76^ using a fifth-order polynomial. A reference melt composition (*F*_r_ = 100%) was chosen at 55 wt.% SiO_2_ and the final (eutectic) melt was constrained at 79 wt% SiO_2_ (red crosses on Supplementary Fig. 6). The relative mass fractions of melt for each data series were then adjusted relative to ref. ^69^ such that the SiO_2_ concentration of the starting melt of each series lies on the best-fit regression line for the whole dataset (Supplementary Fig. 6). SiO_2_ concentrations of glasses from ref. ^75^ were interpolated from those of ref. ^72^ on the basis of the run temperature. The regression yields:

8 $$100 \cdot F_{\mathrm{{r}}} =	\; 91111.54\left( { \pm 1.00} \right) - 7201.176 \cdot {\mathrm{{SiO}}}_2 + 227.5596 \cdot ({\mathrm{{SiO}}}_2)^2\\ 	{ - 3.585154( \pm 0.000010) \cdot ({\mathrm{{SiO}}}_2)^3 + 0.0281282 \cdot ({\mathrm{{SiO}}}_2)^4}\\ 	{ - 0.00008787324 \cdot ({\mathrm{{SiO}}}_2)^5}$$

where SiO_2_ is in wt%. For each modelled path, a starting (i.e., *F*_0_ = 1) concentration of SiO_2_ in the melt at the point of fluid-saturation was randomly selected within the input range of values. Each data series then follows the parameterized calc-alkaline trend (Eq. (8)) as melt fraction decreases. This method effectively simulates parent melts with different initial H_2_O concentration, because less hydrous parents attain H_2_O saturation at higher SiO_2_ concentrations, all other factors being equal. Equation (8) was used to estimate the relative melt fraction of each starting composition $F_{{\mathrm{{r}}}_0}$ (i.e., normalized relative to a hypothetical 100% melt with 55 wt% SiO_2_), and the corresponding relative melt fraction at each step (in the system melt + crystals) was calculated as

9 $$F_{{\mathrm{{r}}}_i} = \frac{{m_{{\mathrm{{melt}}}_i}}}{{m_{{\mathrm{{melt}}}_i} + m_{{\mathrm{{crystals}}}_i}}} \cdot F_{{\mathrm{{r}}}_0}$$

Using $F_{{\mathrm{{r}}}_i}$, the concentration of SiO_2_ in the melt at each step was obtained by solving Eq. (8) using a root-finding algorithm.

### Fluid salinity during incremental degassing

Considering stepwise degassing whereby at each degassing increment (*i*) both crystals and exsolved fluid leave the system, the bulk partition coefficient at each increment is:

10 $$D_{{\mathrm{{Cl}}}_i} =	\; B_i^{\mathrm{{c}}} \cdot \left[ D_{{\mathrm{{Cl}}}}^{\frac{{{\mathrm{{crystals}}}}}{{{\mathrm{{melt}}}}}} \cdot \left( {1 - {\mathrm{{H}}}_2{\mathrm{{O}}}_{{\mathrm{{sat}}}_i} \cdot \frac{{100}}{{100 - {\mathrm{{NaCl}}}_{{\mathrm{{fluid}}}_i}}}} \right) + D_{{\mathrm{{Cl}}}_i}^{\frac{{{\mathrm{{fluid}}}}}{{{\mathrm{{melt}}}}}} \cdot {\mathrm{{H}}}_2{\mathrm{{O}}}_{{\mathrm{{sat}}}_i}\right.\\ 	\left. \cdot \frac{{100}}{{100 - {\mathrm{{NaCl}}}_{{\mathrm{{fluid}}}_i}}} \right] + B_i^{\mathrm{{d}}} \cdot \left[ {D_{{\mathrm{{Cl}}}_i}^{\frac{{{\mathrm{{fluid}}}}}{{{\mathrm{{melt}}}}}}} \right]$$

with $D_{{\mathrm{{Cl}}}}^{\frac{{{\mathrm{{crystals}}}}}{{{\mathrm{{melt}}}}}}$ randomly drawn between 0 and 0.1, to allow for some minor Cl incorporation into hydrous minerals such as apatite, amphibole or biotite. At each step the chlorine concentration in the melt is:

11 $${\mathrm{{Cl}}}_{{\mathrm{{melt}}}_i} = \frac{{{\mathrm{{Cl}}}_{{\mathrm{{melt}}}_{i - 1}}}}{{D_{{\mathrm{{Cl}}}_i} + \frac{{F_i}}{{F_{i - 1}}}\left( {1 - D_{{\mathrm{{Cl}}}_i}} \right)}}$$

12 $${\mathrm{{Cl}}}_{{\mathrm{{fluid}}}_i} = D_{{\mathrm{{Cl}}}_i}^{\frac{{{\mathrm{{fluid}}}}}{{{\mathrm{{melt}}}}}} \cdot {\mathrm{{Cl}}}_{{\mathrm{{melt}}}_i}$$

13 $${\mathrm{{NaCl}}}_{{\mathrm{{fluid}}}_i} = {\mathrm{{Cl}}}_{{\mathrm{{fluid}}}_i} \cdot \frac{{M_{{\mathrm{{Na}}}} + M_{{\mathrm{{Cl}}}}}}{{M_{{\mathrm{{Cl}}}}}}$$

Because, according to our experimental results (Fig. 2), the fluid/melt partition coefficient for Cl (i.e., water + NaCl_fluid_) depends on fluid salinity, at each step this parameter was solved incrementally using Eqs. (10)–(13) until convergence was achieved. Paths for which at least one step resulted in fluid salinity >25 wt% were removed during post-processing because it lies far outside the calibration range of Eq. (1).

### Concentration of Cu in instantaneous fluid fractions

Initial melt Cu concentrations were randomly chosen between 10 and 150 ppm, consistent with the range of Cu concentration in arc andesites and dacites worldwide^44^. The bulk partition coefficient for Cu was calculated in a similar way to Cl, following Eq. (10) adapted by replacing Cl with Cu in the equation. In order to evaluate the effect of sulphide crystallization during degassing (and indirectly the effect of oxygen fugacity and S concentration), the crystal/melt partition coefficient for Cu was randomly chosen between 0.2 and 2.0. Considering a sulphide/melt and a bulk (silicate + oxides)/melt partition coefficient of 2000 and 0.2 for Cu, respectively^77,78^, this corresponds to a sulphide mass fraction of 0–0.09% in the crystallizing assemblage. The fluid/melt partition coefficient for Cu was estimated following the salinity-dependent calibration of Eq. (14) ^17^.

14 $$D_{{\mathrm{{Cu}}}_i}^{\frac{{{\mathrm{{fluid}}}}}{{{\mathrm{{melt}}}}}} = 8.0 \cdot 10^4 \cdot \left[ {\left( {X_{{\mathrm{{NaCl}}}_i}^{{\mathrm{{fluid}}}}} \right)^2 \cdot \left( {X_{{\mathrm{{H}}}_2{\mathrm{{O}}}_i}^{{\mathrm{{fluid}}}}} \right)^{14} \cdot \left( {1 + 180 \cdot X_{{\mathrm{{H}}}_2{\mathrm{{S}}}_i}^{{\mathrm{{fluid}}}}} \right)} \right] + 380 \cdot X_{{\mathrm{{NaCl}}}_i}^{{\mathrm{{fluid}}}} + 0.8$$

assuming $X_{{\mathrm{{H}}}_2{\mathrm{{S}}}_i}^{{\mathrm{{fluid}}}} = 0$ for simplicity. The Cu concentration in the melt and fluid at each step were calculated following variants of Eqs. (11), (12) adapted by replacing Cl with Cu in the equation.

### Aggregated fluid

For each degassing path, we computed the salinity and Cu concentration of the aggregated extracted fluid as a weighted mean of the fluid fractions degassed at each increment along the path:

15 $${\mathrm{{NaCl}}}_{{\mathrm{{aggr.fluid}}}} = \frac{1}{{m_{{\mathrm{{fluid}}}_n}}} \cdot \mathop {\sum}\limits_{i = 1}^n {{\mathrm{{NaCl}}}_{{\mathrm{{fluid}}}_i}} \cdot (m_{{\mathrm{{fluid}}}_i} - m_{{\mathrm{{fluid}}}_{i - 1}})$$

16 $${\mathrm{{Cu}}}_{{\mathrm{{aggr.fluid}}}} = \frac{1}{{m_{{\mathrm{{fluid}}}_n}}} \cdot \mathop {\sum}\limits_{i = 1}^n {{\mathrm{{Cu}}}_{{\mathrm{{fluid}}}_i}} \cdot (m_{{\mathrm{{fluid}}}_i} - m_{{\mathrm{{fluid}}}_{i - 1}})$$

where

17 $$m_{{\mathrm{{fluid}}}_i} = m_{{\mathrm{{water}}}_i} + (m_{{\mathrm{{water}}}_i} - m_{{\mathrm{{water}}}_{i - 1}}) \cdot \frac{{{\mathrm{{NaCl}}}_{{\mathrm{{fluid}}}_i}}}{{100 - {\mathrm{{NaCl}}}_{{\mathrm{{fluid}}}_i}}}$$

### Extraction efficiency

For each path the Cu extraction efficiency was defined as

18 $$\frac{{m_{{\mathrm{{fluid}}}_n} \cdot {\mathrm{{Cu}}}_{{\mathrm{{aggr.fluid}}}}}}{{m_{{\mathrm{{melt}}}_0} \cdot {\mathrm{{Cu}}}_{{\mathrm{{melt}}}_0}}}$$

## Supplementary information

Supplementary Information

Description of Additional Supplementary Files

Supplementary Data 1

## Data Availability

All data used in the parameterizations and modelling can be found in the Supplementary Data file. Raw experimental data can be obtained from the corresponding author on reasonable request. Data used in Figs. 1–4 are also included in the Supplementary Data file.

## Code availability

The R code used to generate the results and figures presented in this paper is available upon reasonable request to the authors. In addition, computation of the chlorine fluid/melt partition coefficient and of the equilibrium composition of fluid and melt have been implemented into a standalone web app called *BubbleMag*: https://cchellem.shinyapps.io/BubbleMag/
