# Supplementary material for: Chemical feedbacks during magma degassing control chlorine partitioning and metal extraction in volcanic arcs
Source: Nat Commun. 2021 Mar 19;12:1774. doi: 10.1038/s41467-021-21887-w (PMC7979762; doi:10.1038/s41467-021-21887-w)
Supplement: Supplementary file 1 — Supplementary Information [file 41467_2021_21887_MOESM1_ESM.pdf]

Chemical feedbacks during magma degassing control chlorine partitioning and metal extraction in volcanic arcs

B. Tattitch[1]*, C. Chelle-Michou[1,2], J. Blundy[1,3] and R. R. Loucks[1,4]

[1]*School of Earth Sciences, University of Bristol, Bristol BS8 1RJ, UK.*

[2]*Institute of Geochemistry and Petrology, Department of Earth Sciences, ETH Zürich, Clausiusstrasse 25, 8092 Zürich, Switzerland.*

[3]*Department of Earth Sciences, University of Oxford, South Parks Road, Oxford OX1 3AN, UK.*

[4] *Centre for Exploration Targeting, School of Earth and Environment, University of Western Australia, Crawley, WA 6009, Australia.*

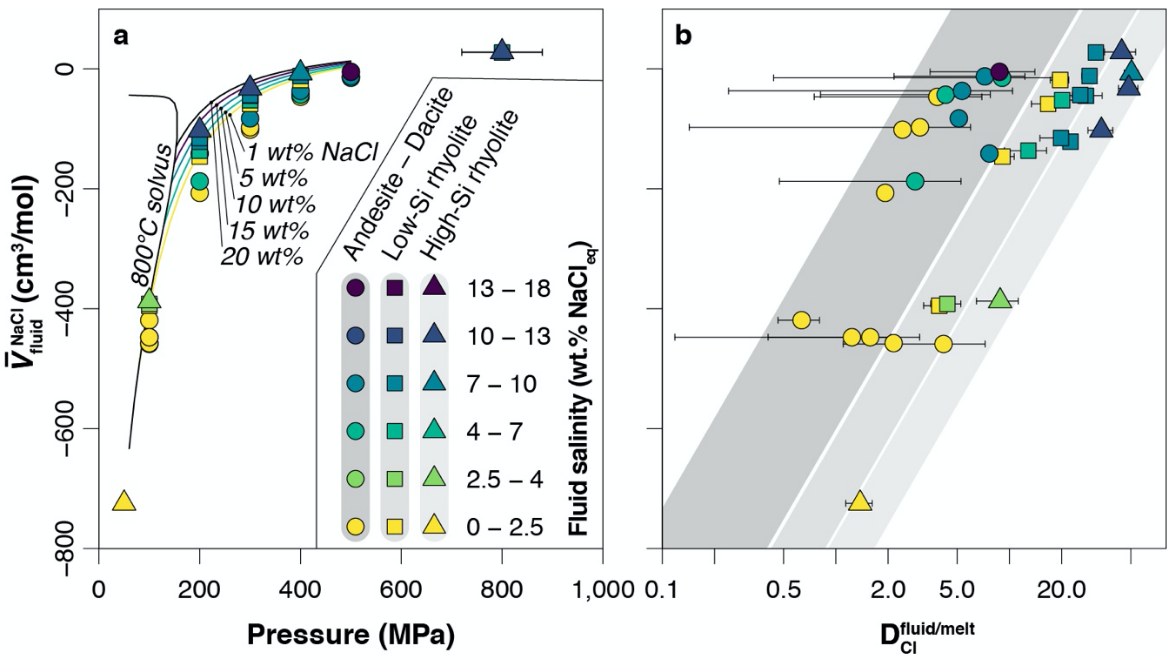

Supplementary Figure 1. Relationship between the calculated apparent partial molar volume of NaCl in the fluid ($\bar{V}_{NaCl}^{fluid}$), and the experimental pressure and $D_{Cl}^{fluid/melt}$. The apparent partial molar volume of NaCl of each point was calculated at the experimental conditions (P and T) according to ref. 60. **a,** With decreasing pressure from ~300 MPa down to 50 MPa the apparent partial molar volume of NaCl in aqueous fluids decreases dramatically. In contrast, as pressure increases away from 300 MPa the fluid undergoes much more limited changes in density and thus $\bar{V}_{NaCl}^{ex}$ shows very little change. Note that the high temperature of andesitic and dacitic experiments (950–1,200 °C) make these points plot below the theoretical curves computed at 800 °C for a range of salinities. **b,** At any given melt and fluid composition, $log(D_{Cl}^{fluid/melt})$ is strongly correlated with $\bar{V}_{NaCl}^{ex}$. This suggests that thermodynamic properties of NaCl in the fluid phase exert the strongest control on $D_{Cl}^{fluid/melt}$ as a function of pressure.

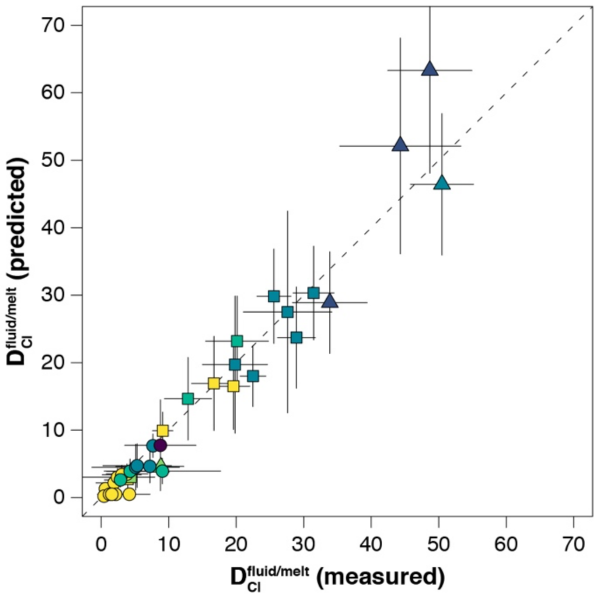

Supplementary Figure 2. Comparison between the measured and the predicted $D_{Cl}^{fluid/melt}$ values for the calibrant dataset using equation (1). Symbol key is the same as in Figure 2.

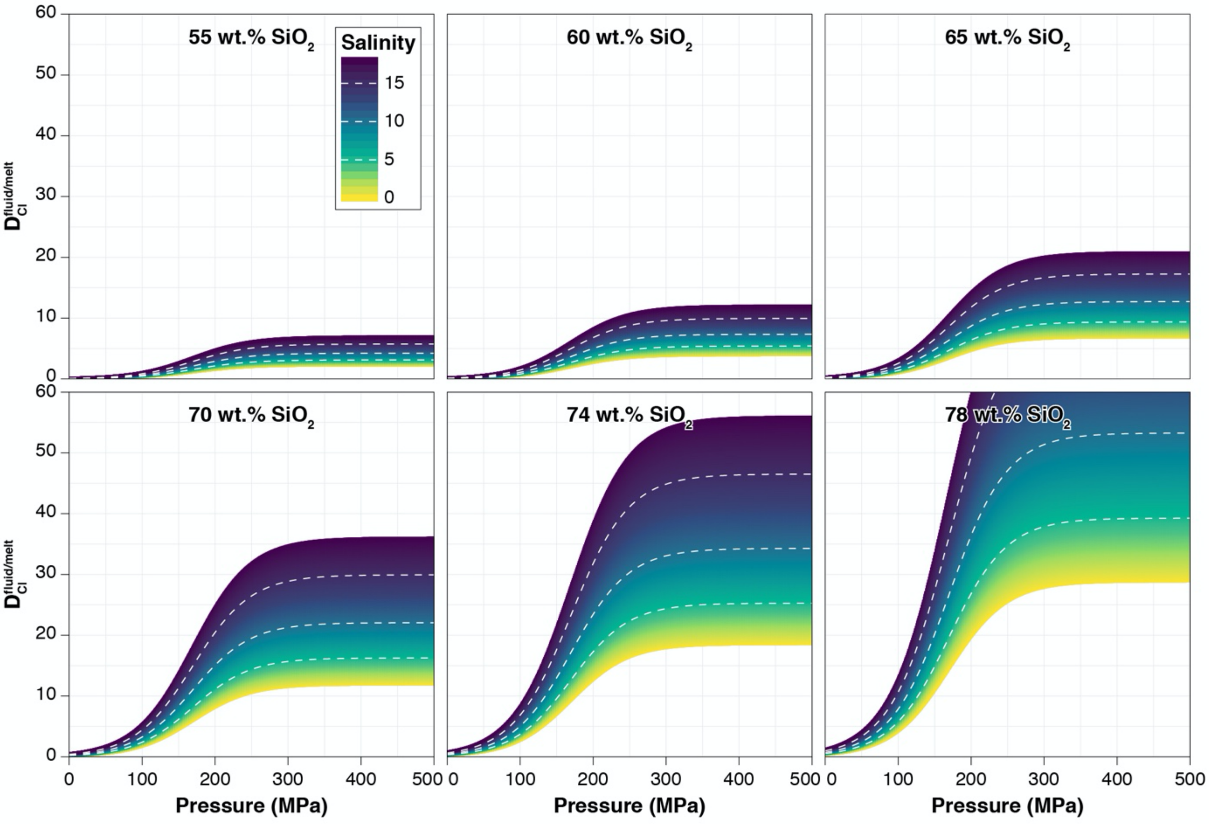

Supplementary Figure 3. Pseudosections of $D_{Cl}^{fluid/melt}$ as a function of pressure and fluid salinity (in wt.% $NaCl_{eq}$) calculated using equation (1) for melts with several fixed SiO_2 concentrations.

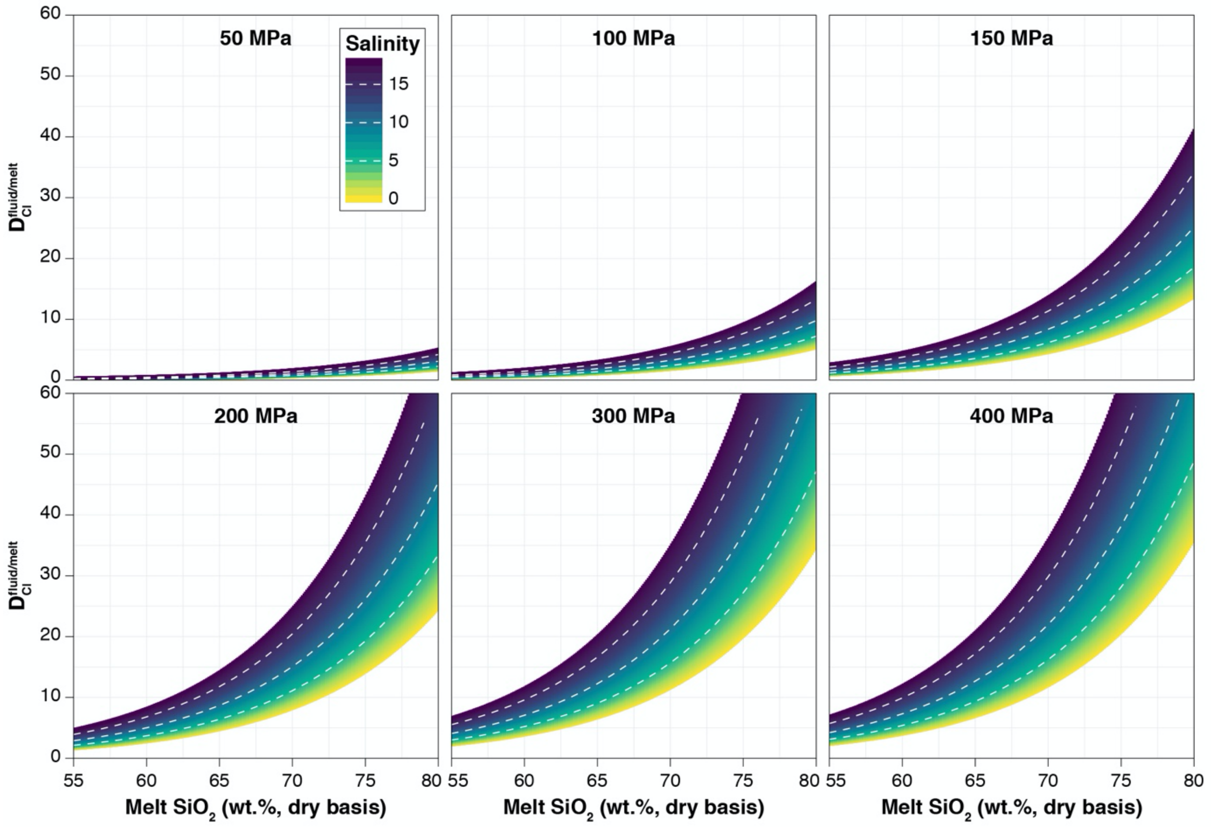

Supplementary Figure 4. Pseudosections of $D_{Cl}^{fluid/melt}$ as a function of melt SiO_2 concentration and fluid salinity (in wt.% $NaCl_{eq}$) calculated using equation (1) at different fixed pressures. No change in the values of $D_{Cl}^{fluid/melt}$ as a function of pressure is observed between 400 and 800 MPa.

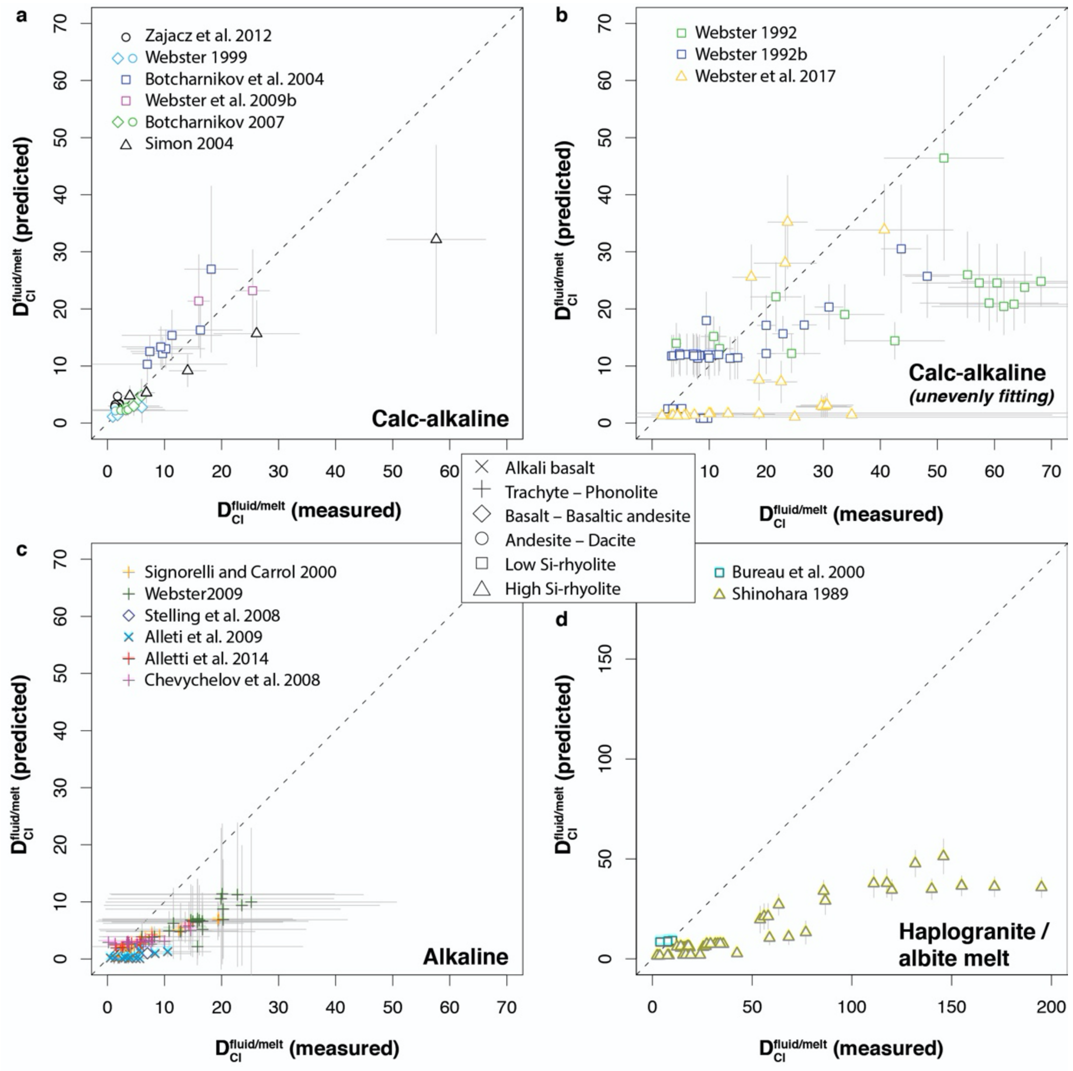

Supplementary Figure 5. Quality of fit comparisons between our parameterization of $D_{Cl}^{fluid/melt}$ and other literature data[18,20,22-25,65-75] of various melt compositions. The parameterization of chlorine partitioning presented in this study is based upon experimental datasets that cover a wide range of pressures as well as utilizing either direct measurement of the melt and fluid or robust mass balance techniques. In addition, we focused on experimental data that is applicable to calc-alkaline differentiation trends typical to arc magmas around the world, notably those most commonly associated with porphyry copper mineralization. Within the existing literature data on Cl partitioning there are numerous studies of high quality that also examine calc-alkaline melt-fluid partitioning, but which examine a more restricted range of degassing conditions. **a,** The majority of these calc-alkaline experimental data are well reproduced by our parameterization of $D_{Cl}^{fluid/melt}$. **b,** Some of the experimental data by Webster et al. are somewhat less-well reproduced, while often showing linear trends away from our calibration indicating that another parameter may be systematically influencing $D_{Cl}^{fluid/melt}$ in these experiments. The role of different melt parameters is more evident and systematic when examining alkaline (**c**) and haplogranite or albitic (**d**) melt compositions. **c** and **d,** show that these datasets also show an offset trend to our calibration indicating that there are likely important melt parameters other than SiO_2 that affect $D_{Cl}^{fluid/melt}$ in melts systems that are not representative of calc-alkaline magmatism. Further characterization of these compositional effects over a wide pressure range is a rich area for future studies.

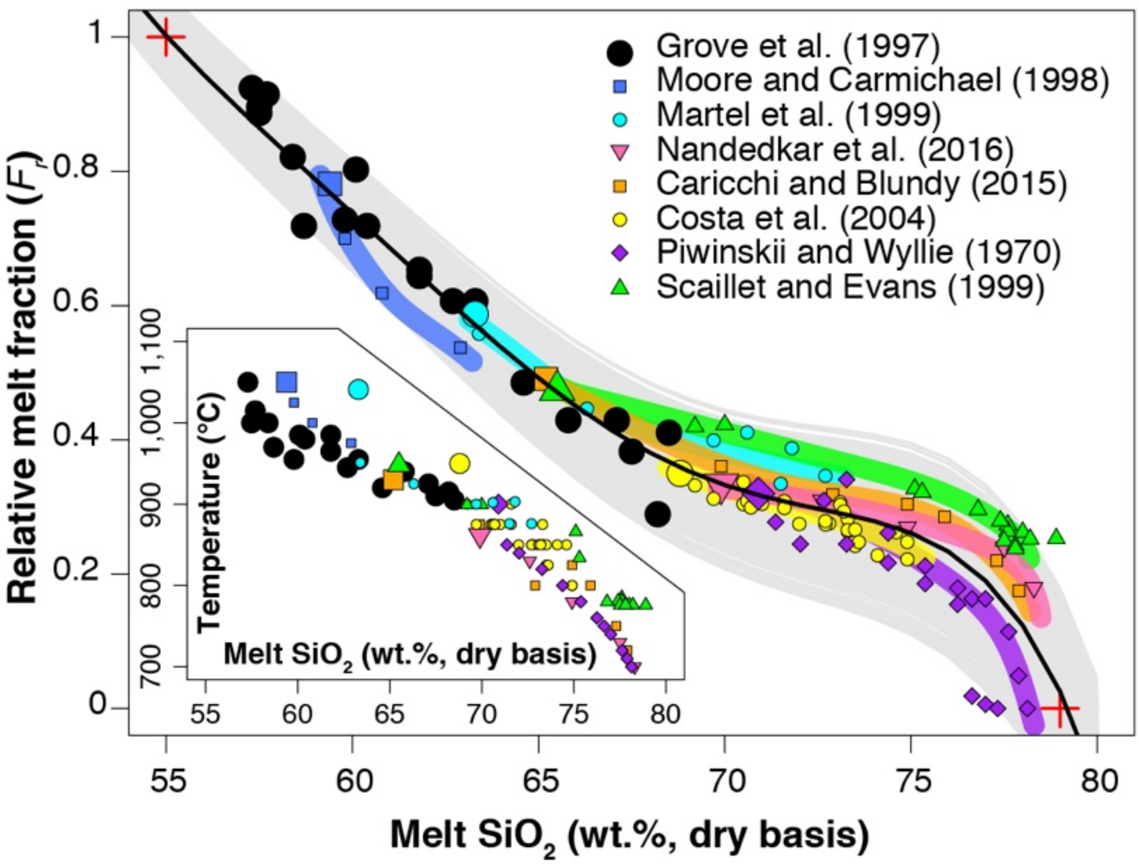

Supplementary Figure 6. SiO_2–relative melt fraction relationship for a suite of water-saturated experiments on calc-alkaline magmas[77-84]. The absolute melt fraction of each experiment was normalized to a reference 100% melt of 55 wt.% SiO_2 (top left red cross). The relative mass fractions of melt for each data series were then adjusted relative to the data series of ref. 77 (black points) such that the SiO_2 concentration of the most primitive melt of each series lies on the best-fit regression line for the whole dataset (black line). The normalized data were regressed using a fifth-order polynomial (black line, equation [8]). The grey lines around the best-fit line represent a series of SiO_2–F_r relationship obtained by randomly varying the constant and the third order coefficient within their assigned uncertainties (see equation [8]) which were included in the model. Inset shows the correlation between melt SiO_2 concentration and temperature for the same dataset.

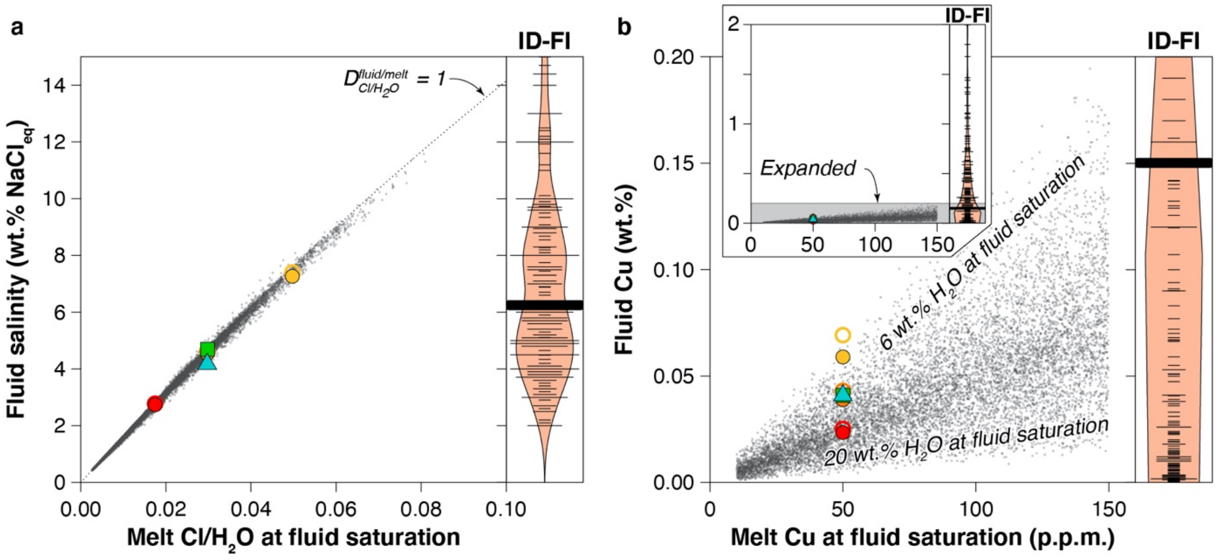

Supplementary Figure 7. Main magmatic controls on the composition of the aggregated fluid. a, The salinity of the fluid is controlled by the mass Cl/H_2O ratio at fluid saturation indicating that essentially all the Cl and H_2O initially present in the melt are degassed. **b,** The Cu concentration in the aggregated fluid logically depends on the Cu concentration in the melt at fluid saturation but may also be diluted or concentrated depending on the amount of magmatic water. The bean plots show the density distribution of the composition of intermediate-density fluid inclusion (ID-FI; data from ref. 38) from intrusive systems for comparison. The thick black lines indicate the median of the population. The inset in **b** shows that the model cannot reproduce the high Cu concentration measured in ID-FI (i.e. the upper half of the measured value). This is probably due to post-entrapment modification by diffusive processes well-documented in low- to intermediate-density fluid inclusions[41,42]. The degassing paths and symbol key are the same as shown in Figure 4.

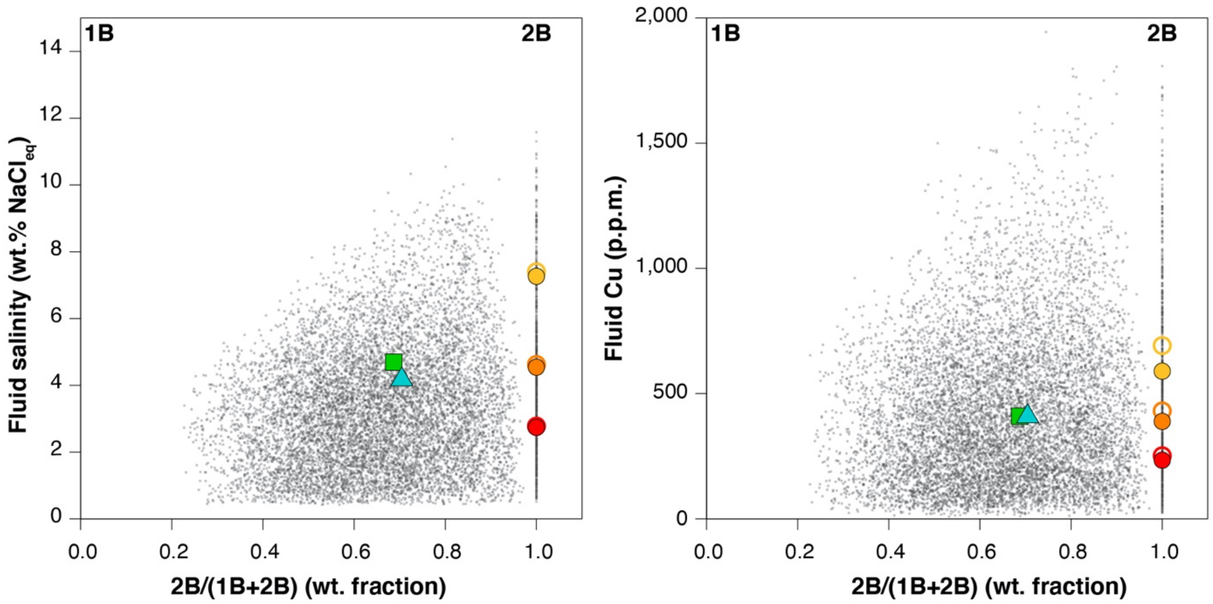

Supplementary Figure 8. Composition of the aggregated fluid as a function of degassing style. The 2B/(1B+2B) parameter corresponds to the ratio between mass of water degassed by second boiling (2B) and the total mass of water degassed by both first and second boiling (1B+2B). It represents a description of the degassing path shown in Figure 4a. The composition of the aggregated fluid is minimally affected by the shape of the degassing path.

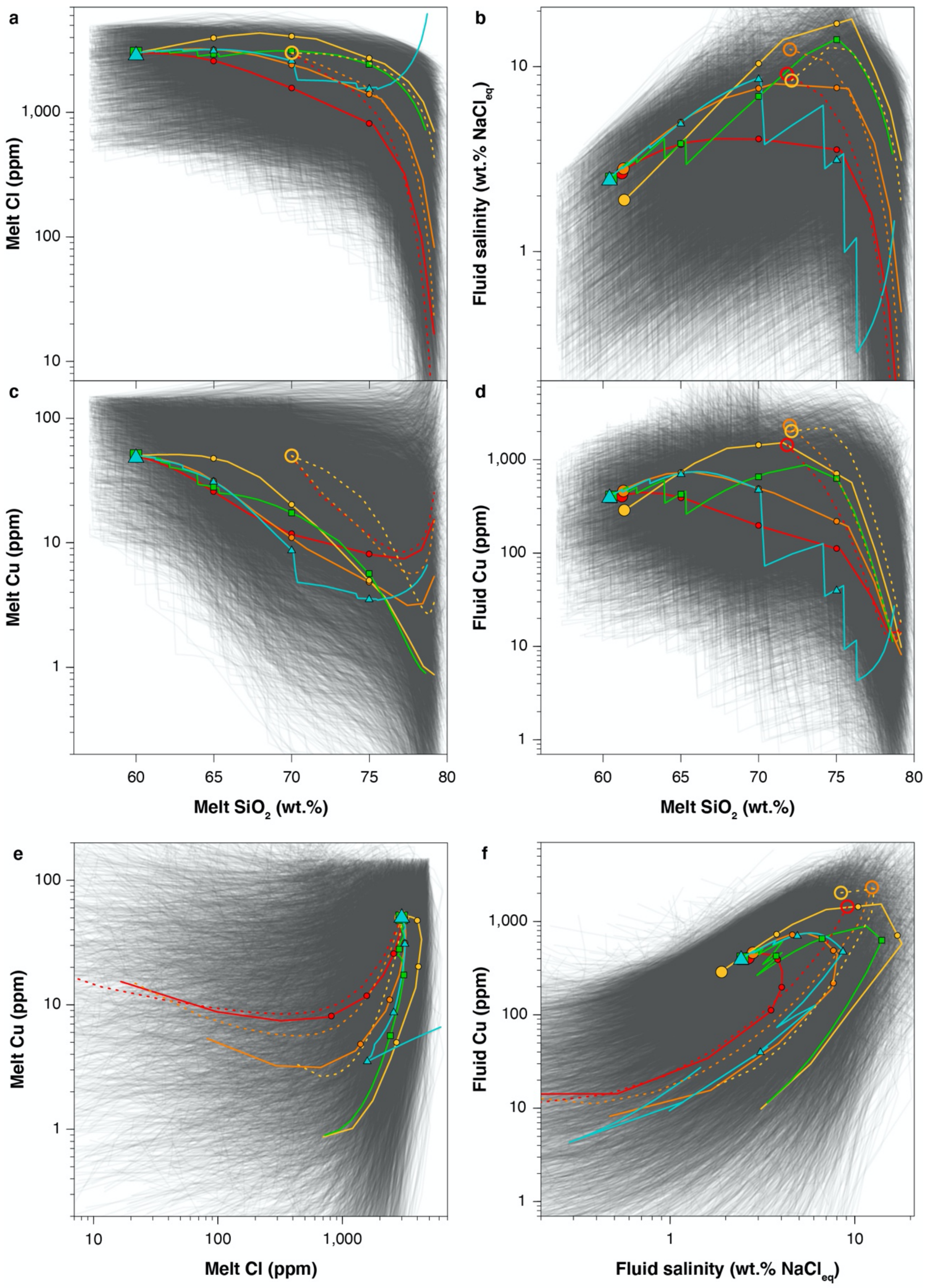

Supplementary Figure 9. Outputs of Monte Carlo simulations illustrating the chemical feedbacks between melt and fluid compositions for 10,000 magma degassing paths. a, c, e, Show the covariation of the instantaneous melt Cl, Cu and SiO_2 concentrations. **b, d, f,** Show the covariation of the instantaneous fluid salinity and Cu concentration, and of the melt SiO_2 concentration. Paths are those shown in Figure 4a. Large coloured symbols indicate the beginning of each path, and smaller coloured symbols are shown for reference at 65, 70 and 75 wt.% SiO_2 in the melt.

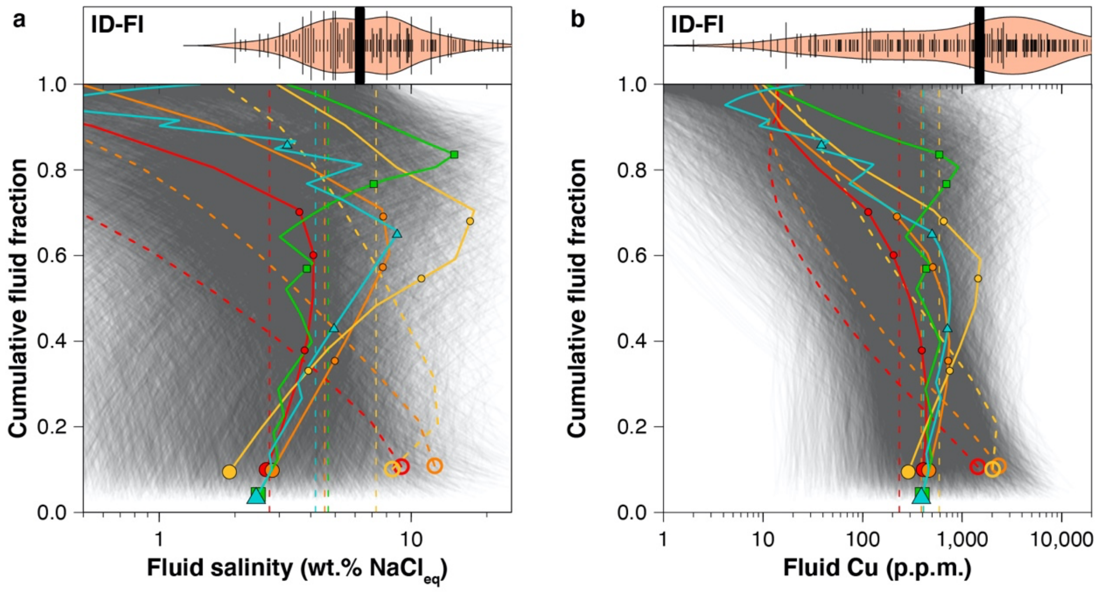

Supplementary Figure 10. Evolution of the composition of the instantaneous fluid as a function of the cumulative degassed fluid mass fraction. a, Salinity of the fluid. **b,** Cu concentration of the fluid. Paths are those shown in Figure 4a. Large coloured symbols indicate the beginning of each path, and smaller coloured symbols are shown for reference at 65, 70 and 75 wt.% SiO_2 in the melt. Vertical dashed lines indicate the composition of the aggregated fluid for the five reference paths. The bean plots show the density distribution of the composition of intermediate-density fluid inclusion (ID-FI; data from ref. 38) from intrusive systems for comparison. The thick black lines indicate the median of the population.

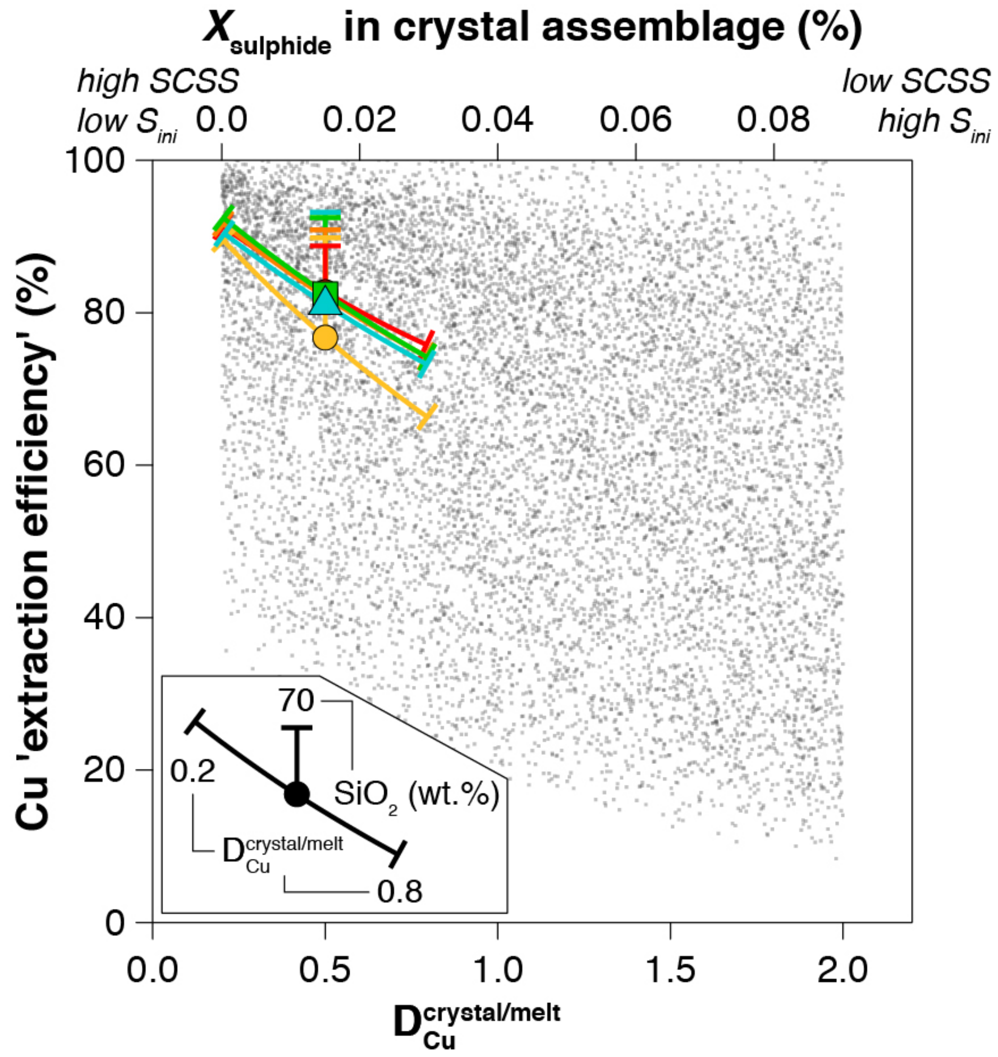

Supplementary Figure 11. Copper extraction efficiency plotted as a function of the bulk $\boldsymbol{D_{Cu}^{crystal/melt}}$. The impact of variation in SiO_2 concentration of the melt at fluid saturation and variable $D_{Cu}^{crystal/melt}$ on Cu extraction efficiency is shown for the coloured example paths. As silicate + oxide minerals have a bulk (silicate+oxides)/melt partition coefficient of 0.2 for most arc magmas[86], variation in $D_{Cu}^{crystal/melt}$ is essentially due to the fractionation of sulphide melt or crystals during degassing. This ultimately reflects the effects of melt composition and fO_2 in modulating the sulphur concentration at sulphide saturation (SCSS). The corresponding fraction of sulphide in the crystallizing mineral assemblage was estimated using a sulphide/melt partition coefficient of 2000 (ref. 85). The degassing paths and symbol key are the same as shown in Figure 4.

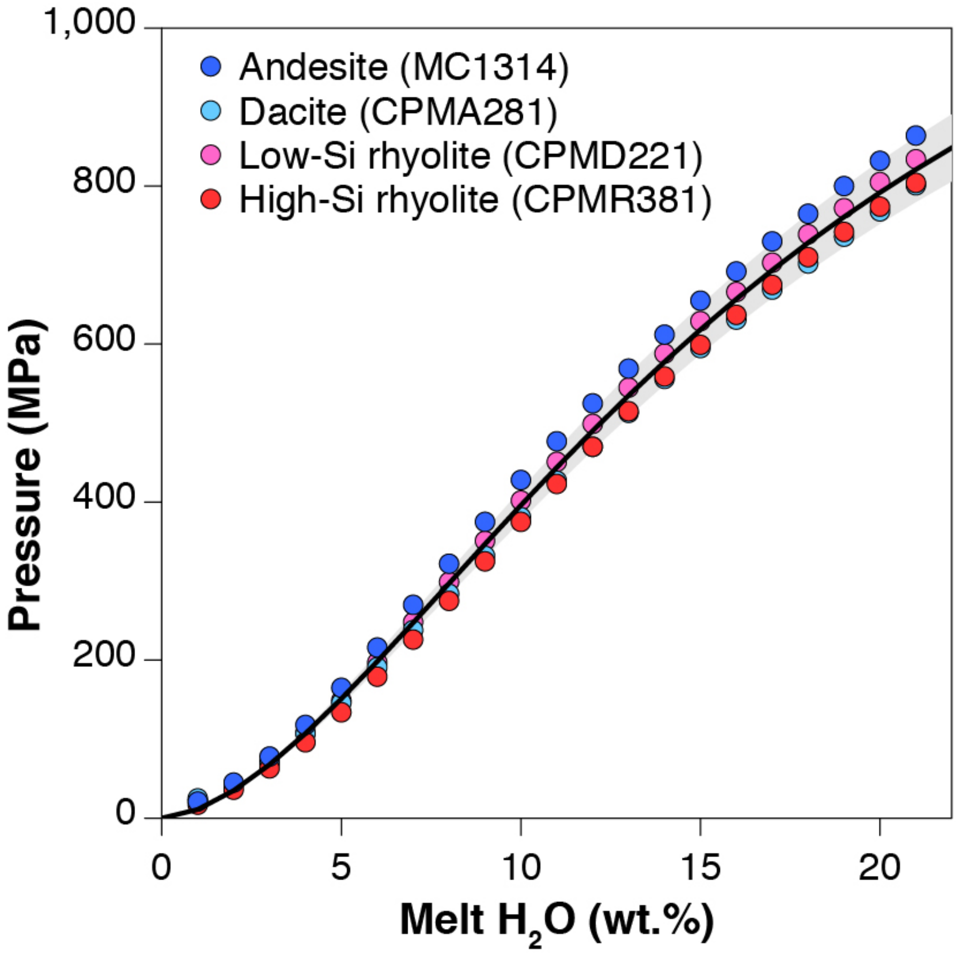

Supplementary Figure 12. Water solubility for melt compositions of the calibrant dataset as a function of pressure. Solubilities were calculated at 800 °C with MagmaSat[32]. The black line and the grey field represent the best fit regression through the points and the 5% relative uncertainty envelop of equation (7), respectively.
