# Supplementary material for: Chemical feedbacks during magma degassing control chlorine partitioning and metal extraction in volcanic arcs
Source: Nat Commun. 2021 Mar 19;12:1774. doi: 10.1038/s41467-021-21887-w (PMC7979762; doi:10.1038/s41467-021-21887-w)
Supplement: Supplementary file 2 — Description of Additional Supplementary Files [file 41467_2021_21887_MOESM2_ESM.pdf]

Descriptions of Additional Supplementary Files

Supplementary Data 1

Description: All data used in the parameterizations and models presented in this work. This includes all available major element data for all melt/glass compositions, fluid compositions (and underlying microthermometry data), experimental conditions, and calculated parameters for newly presented experiments, starting materials and referenced literature values.
